# Determination of Ligand-Binding Affinity (*K*_d_) Using Transverse Relaxation Rate (*R*_2_) in the Ligand-Observed ^1^H NMR Experiment and Applications to Fragment-Based Drug Discovery

**DOI:** 10.1021/acs.jmedchem.3c00758

**Published:** 2023-07-19

**Authors:** Manjuan Liu, Amin Mirza, P. Craig McAndrew, Arjun Thapaliya, Olivier A. Pierrat, Mark Stubbs, Tamas Hahner, Nicola E. A. Chessum, Paolo Innocenti, John Caldwell, Matthew D. Cheeseman, Benjamin R. Bellenie, Rob L. M. van Montfort, Gary K. Newton, Rosemary Burke, Ian Collins, Swen Hoelder

**Affiliations:** † Centre for Cancer Drug Discovery, The Institute of Cancer Research, London SM2 5NG, U.K.; ‡ Division of Structural Biology, The Institute of Cancer Research, London SM2 5NG, U.K.

## Abstract

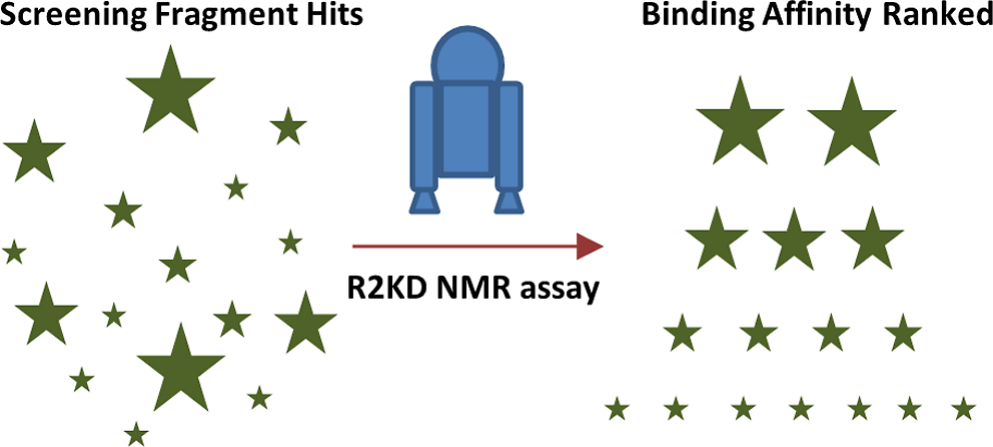

High hit rates from initial ligand-observed NMR screening can make it challenging to prioritize which hits to follow up, especially in cases where there are no available crystal structures of these hits bound to the target proteins or other strategies to provide affinity ranking. Here, we report a reproducible, accurate, and versatile quantitative ligand-observed NMR assay, which can determine *K*_d_ values of fragments in the affinity range of low μM to low mM using transverse relaxation rate *R*_2_ as the observable parameter. In this study, we examined the theory and proposed a mathematical formulation to obtain *K*_d_ values using non-linear regression analysis. We designed an assay format with automated sample preparation and simplified data analysis. Using tool compounds, we explored the assay reproducibility, accuracy, and detection limits. Finally, we used this assay to triage fragment hits, yielded from fragment screening against the CRBN/DDB1 complex.

## Introduction

Advances in molecular biology offer novel protein targets for drug discovery, with up to 10,000 candidates potentially suitable for drug intervention.^1^ Many of these targets act through novel mechanisms and are currently considered challenging to drug. Over the past 20 years, fragment-based drug discovery (FBDD) has risen as an effective strategy, providing successful tool compounds for novel targets, and resulting in the development of new drugs.^2^ By 2021, Erlanson et al. reported six fragment-derived new drugs (Vemurafenib, FDA approved at 2011; Venetoclax, 2016; Pexidartinib 2019; Erdafitinib 2019; Sotorasib, 2021; and Asciminib 2021) against six different targets and a list of 43 molecules in active clinical trials.^3^

The weak affinity (high μM to low mM) of initial fragment hits in FBDD has heralded the innovation of a wide range of biophysical assays.^4^ Among them, the three most popular technologies are nuclear magnetic resonance (NMR), surface plasmon resonance (SPR), and differential scanning fluorimetry (DSF). Each of these approaches have their own advantages and limitations. SPR can provide binding affinity (*K*_d_) information; however, it requires the protein to be physically immobilized to the chip surface, potentially altering the target conformation and/or interfering with the binding pocket. DSF requires the fragment to increase the thermal stability of the target protein, which is not always achievable for weak binding fragments, especially for proteins with high melting points.

Ligand-observed NMR (LONMR) assays are routinely utilized to screen fragments, yielding hits with high μM to low mM binding affinity. During screening, a single ligand concentration is normally employed to qualitatively distinguish binder from non-binder. While this single concentration approach enables screening of 1000–2000 fragments in a reasonable time frame (2–4 weeks) with an acceptable level of protein (20–100 mg) consumption, it is not useful in ranking the affinity of these hits.^5^

NMR-based *K*_d_ determination methods have been published,^6^ and chemical shift perturbation (CSP) using ^15^N-labelled protein is considered the gold standard.^7^ However, CSP is generally restricted to small protein targets (<30 kDa) and is further limited by significant resource costs. *K*_d_ determination by ligand-observed methods such as saturation transfer difference and WaterLOGSY has been reported.^8^ A major limitation of these nuclear Overhauser effect-based experiments is re-binding of the ligand; consequently, measured *K*_d_ values are affected by assay conditions.^9^ A higher protein concentration can potentially result in an artificially higher observed *K*_d_. Line width of NMR signals has also been suggested as an observable parameter to measure *K*_d_. However in practice, line width can be difficult to quantify accurately unless a singlet is clearly observable.^10^

To address these limitations, here we report a quantitative LONMR assay that uses an intrinsic NMR property—transverse relaxation rate *R*_2_—as the observable parameter and determines *K*_d_ values of small molecules in the affinity range of low μM to low mM. In this assay, named as R2KD, we observe the *R*_2_ values of ligands (small molecules) at various concentrations interacting with a single target protein concentration and obtain the *K*_d_ values through curve fitting.

*R*_2_ values of nuclei depend on how fast molecules tumble in solution: small molecules such as fragments tumble very fast, resulting in small *R*_2_ values (e.g., 0.5–2), whereas large molecules such as proteins tumble slowly, resulting in larger *R*_2_ values (e.g., 20–100). When a fragment interacts with a protein, its *R*_2_ value increases, providing a useful observable metric.^11^ In the R2KD assay, we use a routine Carr–Purcell–Meiboom–Gill (CPMG) pulse sequence to experimentally measure *R*_2_ values.

In this study, we examine the basic theory and propose a new mathematical formulation to obtain *K*_d_ using least squares non-linear regression analysis. We design an assay format with automated sample preparation and simplified data analysis. Using tool compounds, we explore the assay accuracy, reproducibility, and detection limits. With examples, we highlight key factors that affect the assay result. Finally, we outline an application of this assay in the triage of fragment hits and in the calculation of *K*_d_ values for selected ligands.

## Theory

**Scheme 1:**
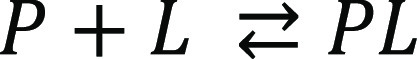
One Site Reversible Binding Process

Considering that the aim was to determine the binding affinity of small molecule fragments to target proteins in early fragment hit discovery programs, a simple one site reversible binding process was assumed. The dynamic equilibrium could be described by Scheme 1, where P represents the protein, L represents the small molecule ligand, and PL represents the protein–ligand complex.

At thermodynamic equilibrium, the binding dissociation constant, *K*_d_, can be defined by eq 1.^12^Eq 1 can be expressed by experimentally controllable variables, *P*_T_ (total protein concentration) and *L*_T_ (total ligand concentration). The concentrations *P*_T_, *L*_T_, [*L*], [*P*], and [PL] are related by eqs 2 and 3. [P] is the free protein concentration, [L] is the free ligand concentration, and [PL] is the protein–ligand complex concentration, or protein-bound ligand concentration. Substituting eqs 2 and 3 into eq 1 gives eq 4, which can be rearranged to quadratic eq 5.

**Figure 1:**
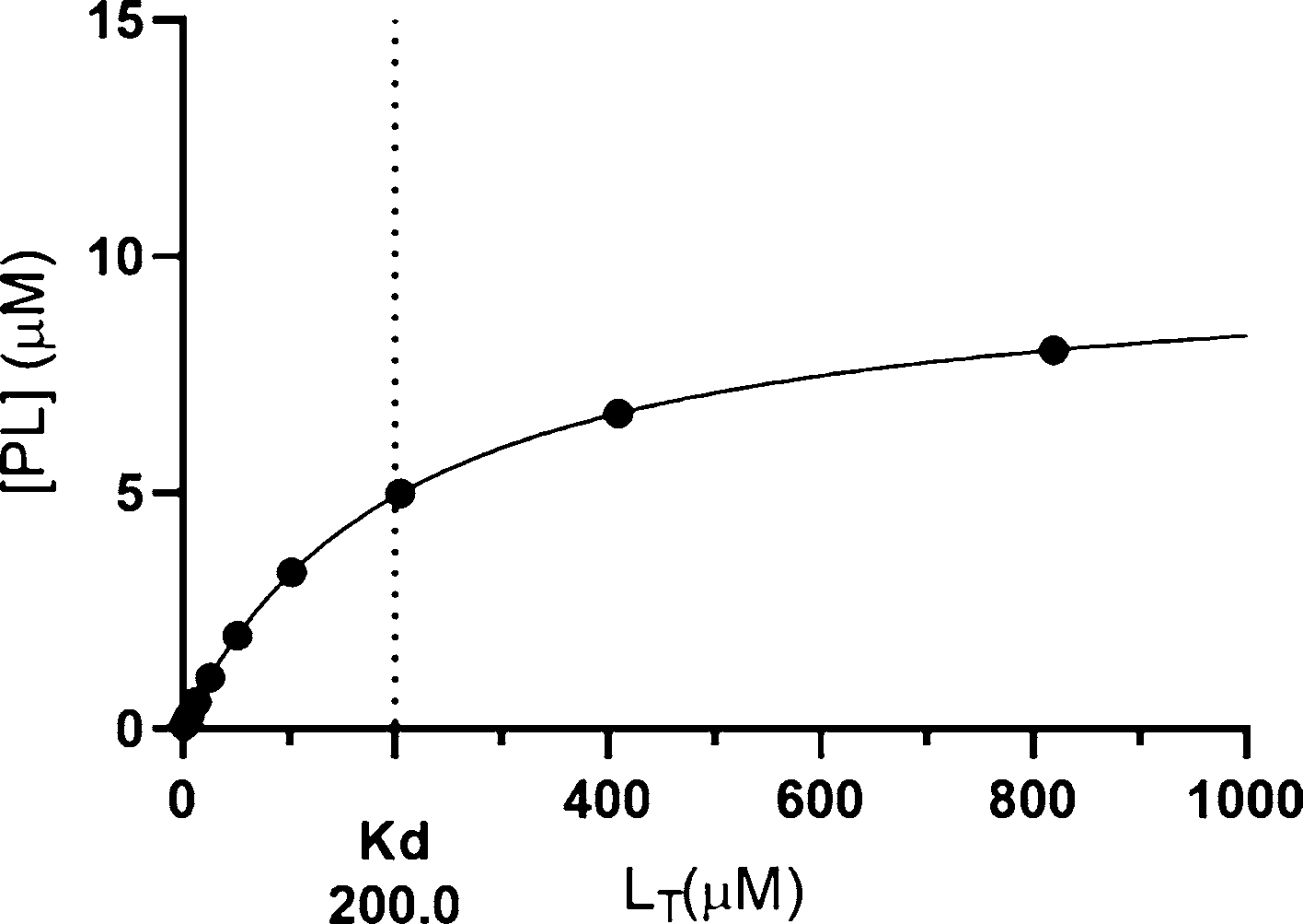
Simulation of bound ligand concentration [PL] as a function of increasing total ligand concentration L_T_ using eq 6 and with P_T_ of 10 μM and *K*_d_ of 200 μM.

A solution to this quadratic equation is eq 6, which allows *K*_d_ determination by non-linear regression if we vary *L*_T_. This equation accommodates a ligand depletion scenario since the [*L*] was not assumed to be *L*_T_. Eq 6 is graphed in Figure 1 using conditions commonly seen in fragment-based ligand NMR experiments. In this study, a linear *x*-axis scale was used as it is more suited to visualize results for fragments with *K*_d_ in the range of μM to low mM.
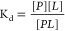
1
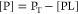
2
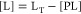
3
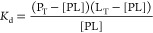
4
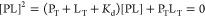
5
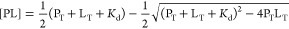
6

Since in LONMR experiments, bound ligand concentration [PL] cannot be observed directly, a suitable observable parameter was required to substitute [PL]. For a weak binding event, and under high ligand excess condition, we could substitute [PL] with *R*_2_ using the mathematical manipulations outlined below (7).

By designing the binding experiment such that the ligand–protein ratio was much higher than 1, i.e., L_T_/P_T_ ≫ 1, the free ligand fraction ρ_F_ becomes much higher than the bound ligand fraction ρ_B_, i.e., ρ_F_ ≫ ρ_B_. Under such conditions, the observed transverse relaxation rate *R*_2,obs_ could be expressed using the Swift-Connick formula, as shown in eq 7, where *R*_2,obs_ is the observed transverse relaxation rate, ρ_F_ = [L]/L_T_ is the free ligand fraction, ρ_B_ = [PL]/L_T_ is the bound ligand fraction, *R*_2F_ is the transverse relaxation rate of the ligand in the free state, *R*_2B_ is the transverse relaxation rate of the ligand in the bound state, *K*_ex_ is the ligand exchange rate between free and bound state, ΔΩ = ω_B_ – ω_F_ is angular precession frequency difference of the ligand in bound and free states, in practice, ΔΩ = 2π(δ_B_ – δ_F_), where δ_B_ and δ_F_ are the chemical shift in Hz. For a weak binding event, the ligand exchange rate was in the range 1000 < *K*_ex_ < 100,000 S^–1^. As a result, the formula could be further simplified to eq 8 according to Peng et al., who gave a detailed explanation for this simplification in their 2004 paper.^13^

For our study, we rewrote eq 8 to eq9 with ρ_F_ and ρ_B_ expressed in terms of [PL], [L], and L_T_. In the instance of a weak binding event, the term  can be approximated to zero, thus allowing the total protein ligand concentration [PL] to be expressed as shown in eq 10.

Combining eq 10 with eq6, we obtained the eq 11, which could be processed in Graphpad Prism to obtain a ligand *K*_d_ value from non-linear regression curve fitting to the experimental data. *R*_2,obs_ was measured experimentally using individual samples containing differing ligand concentrations but identical protein concentrations. *R*_2F_ was measured using sample containing only ligand. In practice, we first used eq 13 to calculate the value of the quantity *y* in Microsoft Excel and then substituted the first term in eq 11 with the factor α as shown in eq 14. Finally, we used Prism to perform non-linear regression with eq 12 which produced fitted values for both *K*_d_ and the factor α.
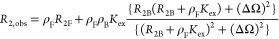
7
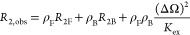
8
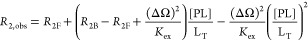
9
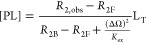
10
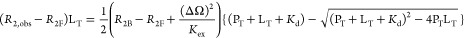
11
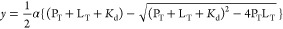
12
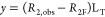
13
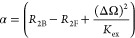
14

## Results and Discussion

### Assay Format and Sample Preparation

Four aqueous stock solutions were prepared in Eppendorf Safe-Lock tubes (Cat no. 0030120094) by manual pipetting: (1) ligand aqueous stock solution was prepared by adding 50 mM ligand DMSO-*d*_6_ solution to aqueous buffer; (2) dimethyl sulfoxide (DMSO) aqueous stock solution was prepared by adding the same volume of DMSO-*d*_6_ as ligand DMSO-*d*_6_ solution to aqueous buffer; (3) protein aqueous stock solution was prepared by adding protein solution to aqueous buffer; and (4) aqueous solution was the buffer used in the assay.

**Figure 2:**
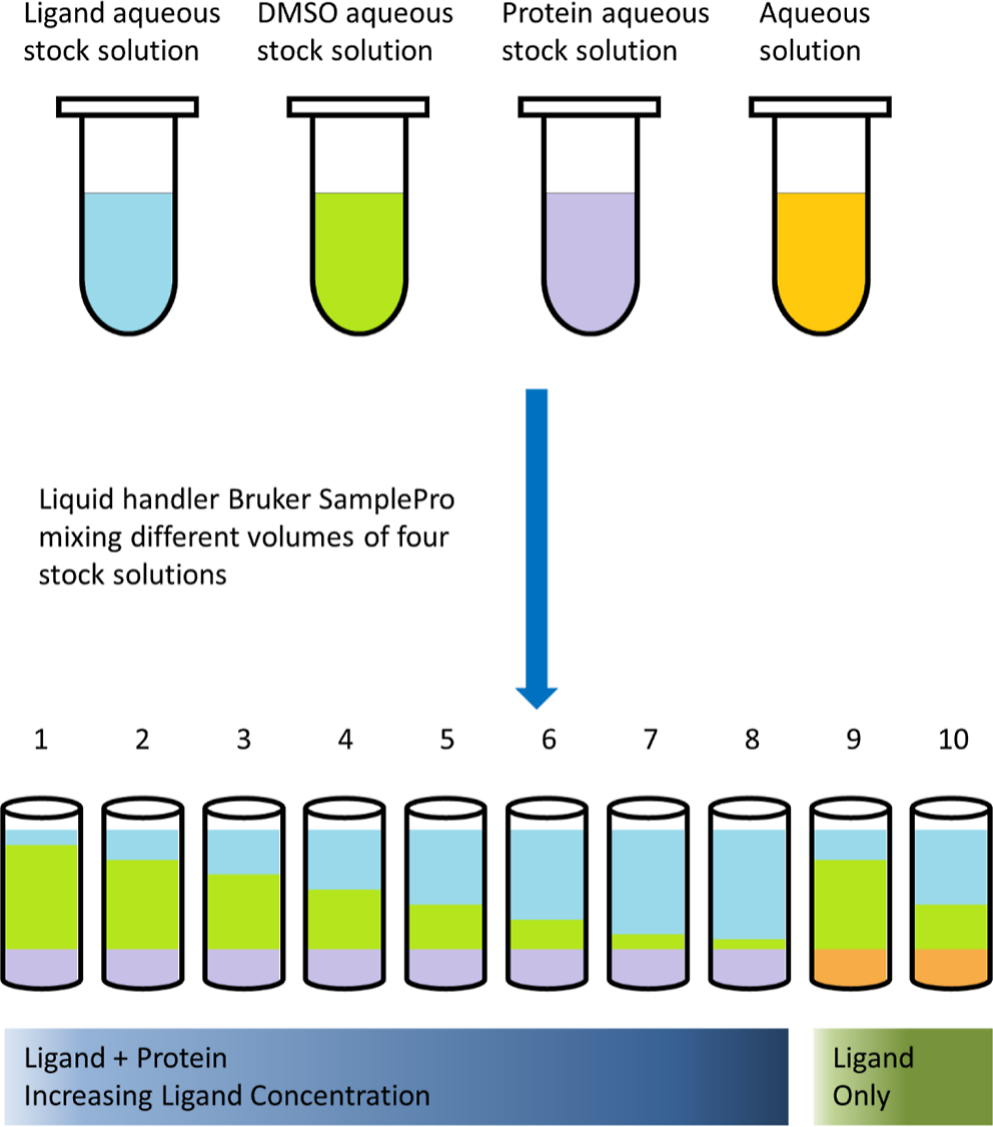
Schematic drawing showing how samples were prepared for R2KD assay.

Samples were prepared on a Bruker SamplePro-Tube liquid handler by mixing different volume of the four aqueous stocks in 96-well microplates (Greiner 650201) and then transferred to 3 mm NMR tubes (Figure 2). 10 samples were prepared: samples 1–8 containing increasing ligand concentration (maintaining constant protein concentration); sample 9 and 10 containing only ligands at two different concentrations (protein absent). These two samples served as control samples to obtain the *R*_2_ values of free ligand. The final percentage of DMSO-*d*_6_ in all samples were identical to minimize possible impacts of DMSO-*d*_6._

### *K*_d_ Determination Demonstrated Using the BCL6 BTB Domain Ligand CCT365133

**Figure 3:**
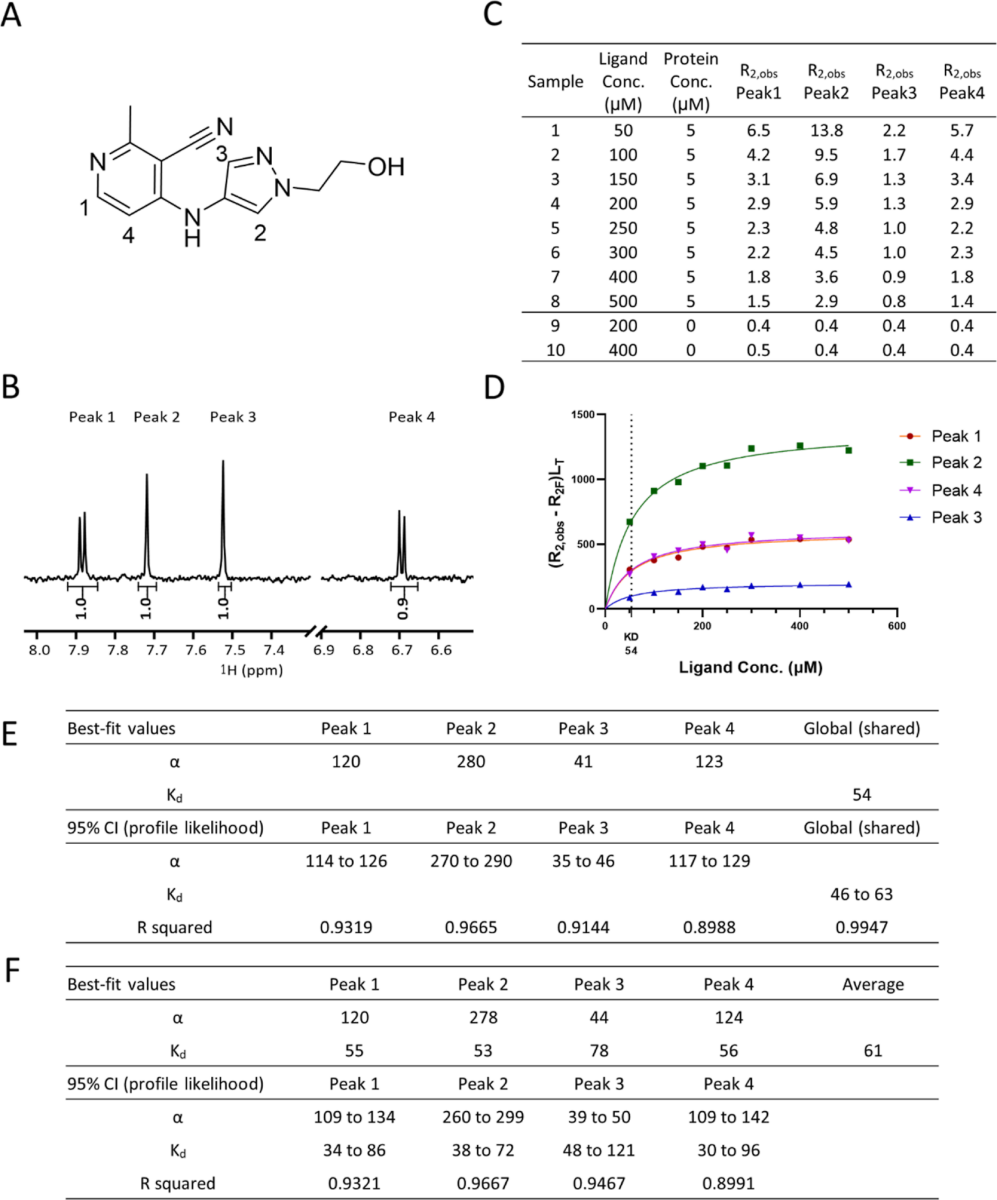
*K*_d_ determination using tool compound CCT365133 against BCL6 BTB. (A) Compound structure, (B) aromatic region of ^1^H-NMR spectrum of CCT365133. (C) *R*_2_ values for all samples (D) *K*_d_ curve was fitted for CCT365133 using *R*_2_ values of four peaks. (E) Graphpad Prism non-linear regression analysis result using eq 12 with global *K*_d_ fitting algorithm. (F) Prism non-linear regression analysis result using eq 12 with *K*_d_ fitted using individual peaks.

Here, we used compound CCT365133 (Figure 3A) to demonstrate how *K*_d_ value could be determined using the R2KD assay. CCT365133 was discovered in our in-house drug discovery program^14^ and determined to interact with the BCL6 BTB domain with K_i_ 50 μM as determined by a TR-FRET binding assay, the assay detail has been published elsewhere.^15^ BCL6 is a transcriptional repressor and has been reported as a potential target for cancer drug therapy.^16^

Figure 3 shows the R2KD assay data used to obtain the *K*_d_ curve for CCT365133. This data was obtained from eight samples containing ligand (CCT365133) at concentrations of 50–500 and 5 μM BCL6 protein as well as from two samples containing ligand only at concentrations of 200 and 400 μM (Figure 3C). The *R*_2_ values of four aromatic ^1^H-NMR signals (Figure 3B) were experimentally determined (Figure 3C) and (*R*_2,obs_ – *R*_2F_) * L_T_ values were calculated using Excel and used as *y*-axis values in Prism, while ligand concentrations were plotted as the *x*-axis values (Figure 3D). All four NMR peaks were used to fit a global *K*_d_ with non-linear regression using a customized equation (see Experimental Section), while α values were fitted as an individual parameter for each peak (Figure 3E) as α values differ depending on the individual proton’s environment. While it was possible to fit *K*_d_ values using individual NMR peaks and then average the values (Figure 3F), we found using a global fitting algorithm increased robustness of curve fitting and better accommodated outliers in the data. The goodness of fit was judged by 95% confidence interval (CI) (profile likelihood) values and *R* squared value. The global *K*_d_ obtained here is close to the K_i_ value (54 vs 50 μM) although from our accuracy study, the differences can be larger (see section “Assay Accuracy”).

### Assay Accuracy

**Figure 4:**
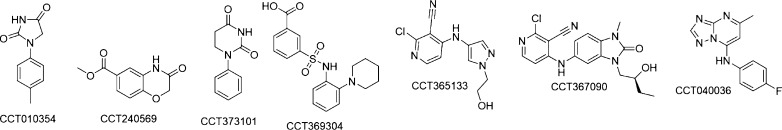
Chemical structures of compounds used in accuracy and reproducibility tests.

To determine the accuracy of *K*_d_ measured using the R2KD assay, we tested seven small molecule ligands (Figure 4) against three protein targets (BCL6, CRBN/DDB1 complex, and ERAP1) and compared their *K*_d_ values from the R2KD assay with known *K*_i_ values (Table 1) obtained in biochemical binding assays^15,17,18^ or *K*_d_ value from the SPR binding assay.^19^ We found good agreements between the *K*_d_ values and the *K*_i_ values, with most of them less than two-fold difference from those determined from biochemical or biophysical assays. These results from three structurally distinct proteins also demonstrated the versatility of the R2KD assay. We found the assay setup to be simple and positive controls unnecessary, making the approach suitable for new and challenging targets.

**Table 1:** *K*_d_ Value Comparison between R2KD Assay and Bioassays

compound	target	target MW	*K*_d_ (μM)	SD (μM)	*K*_i_ (μM)	SD (μM)
CCT010354	^Δ39^CRBN/^ΔBPB^DDB1 complex	141 kDa	156	45	294	154
CCT240569	^Δ39^CRBN/^ΔBPB^DDB1 complex	141 kDa	88	16	140	94
CCT373101	^Δ39^CRBN/^ΔBPB^DDB1 complex	141 kDa	12	10	10	3
CCT369304	ERAP1	111 kDa	196	58	33	14
CCT365133	BCL6 BTB	29 kDa	51	8	50	ND
CCT367090	BCL6 BTB	29 kDa	7	4	4	0.4
CCT040036	BCL6 BTB	29 kDa	1051	128	1500^a^	ND

a Value from SPR assay. *ND: not determined.

### Assay Reproducibility

For these seven compounds, the R2KD assay was repeated three times with sample preparation on separate dates to assess the assay’s reproducibility under optimized assay conditions. The assay was observed to have good reproducibility: compounds with affinity ranged from high μM to low mM had standard deviation (SD) less than 50% of average *K*_d_ value; for compounds in the affinity range between 1 and 20 μM, the error increased to 80% of average *K*_d_ value. As the purpose of this assay is to assess initial, typically weak binding fragment hits, this reproducibility was deemed satisfactory in the relevant affinity range.

### Assay Detection Limit

Based on the theory of the R2KD assay, this approach is best suited to detect low μM to low mM affinity, when the ligand exchanges rapidly between bound and free states. In practice, two main factors limit the detection range: NMR instrument sensitivity and ligand aqueous solubility.

**Figure 5:**
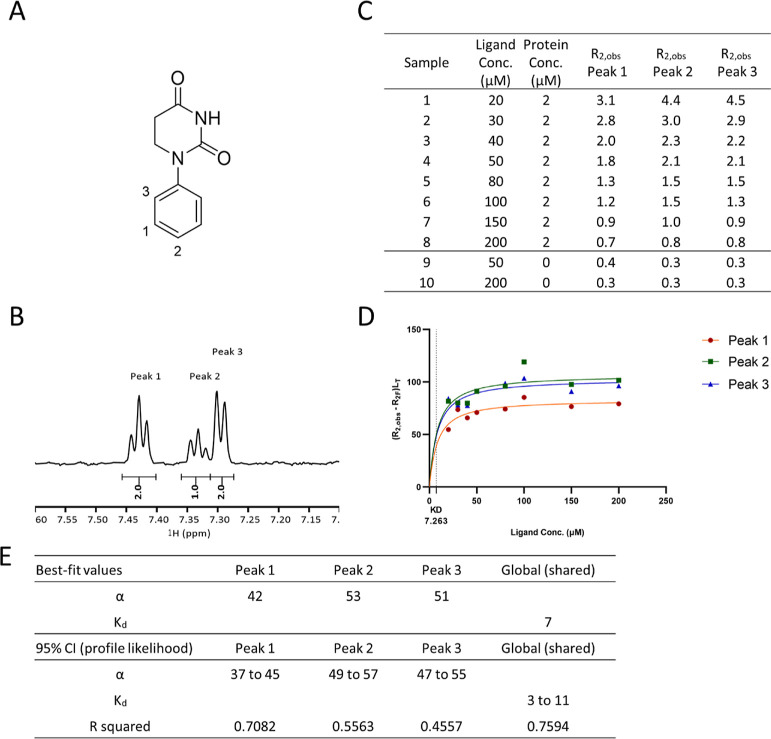
Example of R2KD assay for *K*_d_ less than 10 μM binding to the CRBN/DDB1 complex. (A) Compound structure CCT373101, (B) aromatic region of ^1^H-NMR spectrum of CCT373101, (C) *R*_2_ values for all samples, (D) *K*_d_ curve was fitted for CCT373101 using *R*_2_ values of three peaks, and (E) Graphpad Prism non-linear regression analysis result using eq 12 with global *K*_d_ fitting algorithm.

For the NMR system used in this study (600 MHz with TCI-CryoProbe), we found that 20 μM ligand concentration was the lowest concentration that generated sufficient signal-to-noise level in 1 h for *R*_2_ measurements. This limited the lowest affinity we can accurately measure. In the R2KD assay, *K*_d_ is a fitted parameter value from non-linear regression, so it is possible to obtain *K*_d_ even if its value is outside the experimental concentration range, as demonstrated using CCT373101 (Figure 5). Although a lack of data points could compromise accuracy of the fitted *K*_d_ value with wider 95% CI (Figure 5F), results showed that compounds with around 10 μM *K*_d_ could still be measured with satisfactory accuracy and reproducibility (Table 1). We also observed that specific binding was clearly indicated as *R*_2_ values increased several fold when ligand concentration reduced gradually (Figure 5C). In conclusion, 10 μM was deemed to be the lower detection limit for this assay.

**Figure 6:**
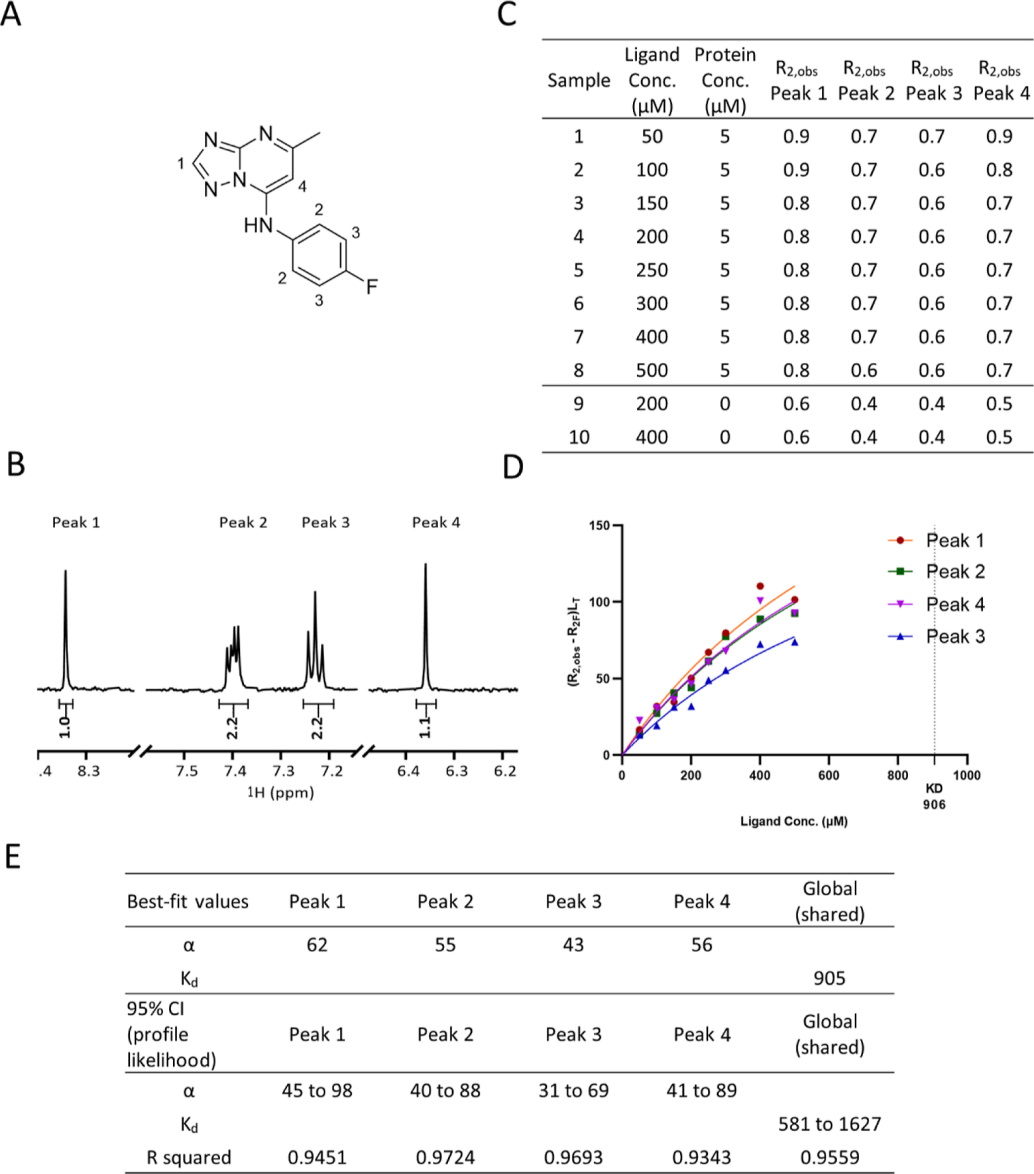
Example of R2KD assay for *K*_d_ greater than 1 mM binding to BCL6. (A) Compound structure CCT040036, (B) aromatic region of ^1^H-NMR spectrum of CCT040036, (C) *R*_2_ values for all samples, (D) *K*_d_ curve was fitted for CCT040036 using *R*_2_ values of four peaks, and (E) Graphpad Prism non-linear regression analysis result using eq 12 with global *K*_d_ fitting algorithm.

For the upper detection limit, as with other assays, small molecule aqueous solubility is a key factor limiting the largest *K*_d_ value detectable. In our experience, a high proportion of fragments have aqueous solubility less than 500 μM, as measured by qHNMR in PBS buffer at pH 7.4, resulting in a near straight line observed within this concentration range for ligands with *K*_d_ higher than 1 mM. Lacking data points from higher ligand concentrations reduced the accuracy of curve fitting. In our tests, the largest *K*_d_ we can determine accurately is around 1 mM using compound CCT040036 binding to BCL6 BTB (Figure 6). However, for soluble fragments like acids or carbohydrates, the upper detection limit can be higher.

To conclude, the R2KD assay is most suited to test compounds with affinity in the 10 μM to 1 mM range.

During the process of developing the R2KD assay, we have noticed several factors that impacted assay performance. The practical implications of such factors are discussed below.

### *R*_2_ Measurement

**Figure 7:**
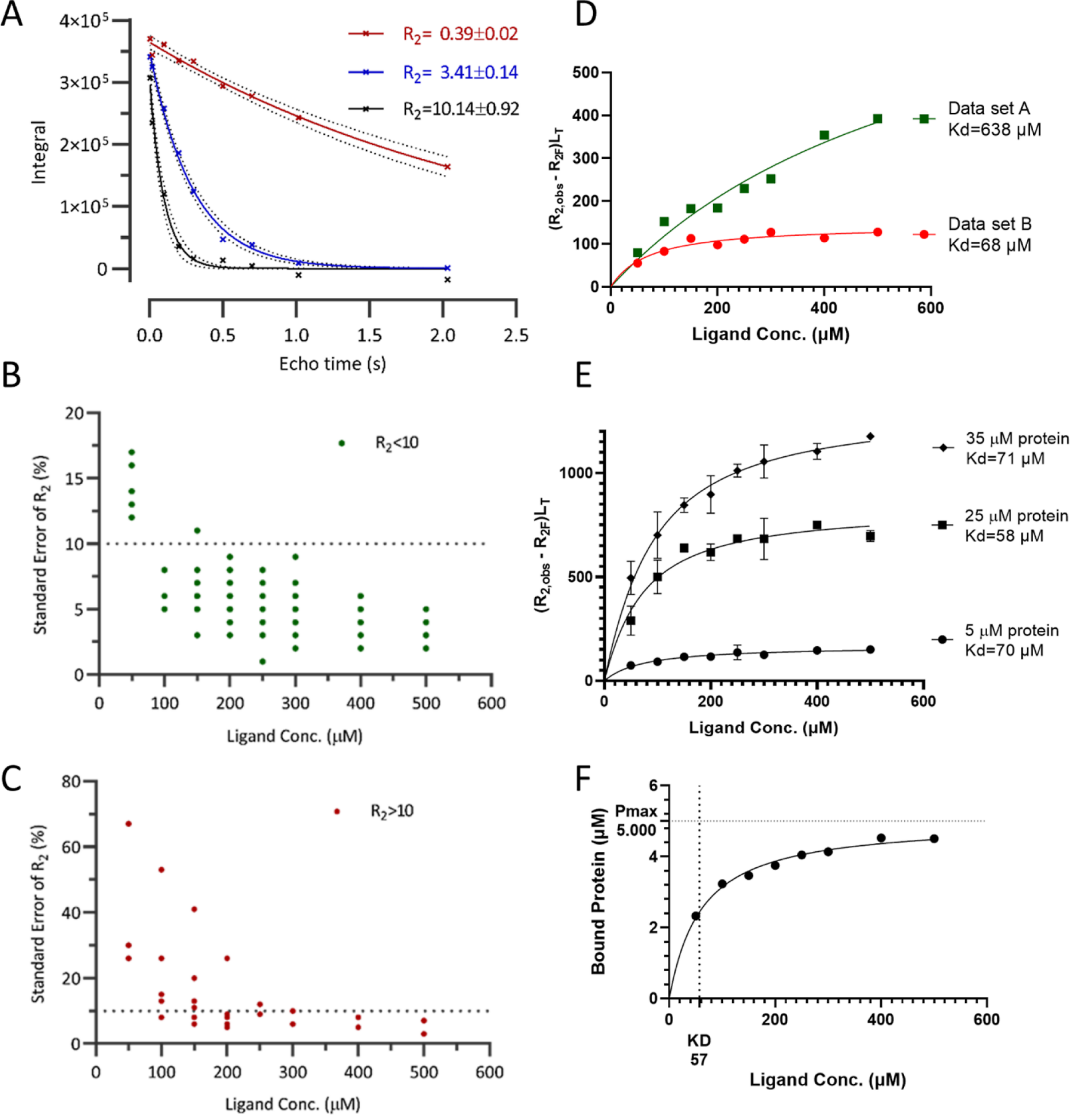
(A) *R*_2_ determination using nonlinear regression with mono-exponential delay equation *I* = *I*_0_exp(*tR*_2_) method. Spin-echo time points were evenly distributed to capture *R*_2_ values between 0.3 and 10 with error less than 10%. (B) Standard error distribution analysis shows for different ligand concentration, *R*_2_ errors were kept low when *R*_2_ values were less than 10. (C) Standard error distribution analysis shows *R*_2_ errors became too high at concentrations lower than 250 μM when *R*_2_ values were greater than 10. (D) Example shown of impact of using ligand beyond its aqueous solubility. Weaker *K*_d_ value was yielded from curve fitting using data set A which simulated situations where compound concentrations plateaued due to limited aqueous solubility. (E) *K*_d_ curves from different protein concentrations showing similar *K*_d_ obtained using peak 3 of CCT365133 whose structure and ^1^H-NMR spectrum are shown in Figure 3. (F) Simplified graphical presentation of *K*_d_ curve fitting using calculated bound ligand concentration [PL]. All figures in Figure 7 were generated using CCT365133 against BCL6 BTB.

To successfully determine *K*_d_, it is essential to know how precisely the *R*_2_ value can be measured, to distinguish changes in *R*_2_ due to interaction with protein from changes due to experimental variation. In this study, *R*_2_ was determined using non-linear regression with mono-exponential delay equation *I* = *I*_0_exp(*tR*_2_) (Figure 7A) using a CPMG pseudo-2D NMR experiment with water suppression. In optimizing the NMR experiment, we aimed to determine *R*_2_ with standard error less than 10% of the *R*_2_ value while minimizing NMR experiment acquisition time to within 1 h for the lowest concentration sample.

We considered that the key factors for the *R*_2_ NMR acquisition experiment were the number of different spin-echo times, how to distribute these spin-echo times, and the total scan number. Ideally, all the relaxation time points should be evenly distributed on the curve with enough data points to give satisfactory resolution for the mono-exponential curve fitting. For samples with low ligand concentrations (e.g., 50 μM), more scan numbers were acquired to allow the software to integrate the peak area under the NMR signals accurately. After some initial analysis, we set up an *R*_2_ experiment using nine different spin-echo times (4, 20, 100, 200, 300, 500, 700, 1000, and 2000 milliseconds) and scan numbers ranging from 8 to 32.

We tested if these parameters were appropriate by analyzing results from 30 *R*_2_ experiments with different ligand protein ratios. We found that when *R*_2_ values were less than 10 (Figure 7B), standard errors were less than 10% for concentrations greater than 50 μM, which was within our intended range. For samples at a concentration of 50 μM, standard errors were between 10 and 17%, higher than we aimed for but still acceptable for our purpose. It was possible to reduce the standard error by increasing NMR experiment scan numbers further, but this required experimental time beyond what was considered routinely practical in our laboratory. However, when *R*_2_ values were greater than 10 (Figure 7C), standard errors were greater than 20% for several concentrations. It may require trial and error to identify assay conditions to avoid *R*_2_ values greater than 10. Instead of changing the *R*_2_ experimental protocol, we opted to either exclude these values when we performed the non-linear regression or to reduce protein concentration to decrease *R*_2_ values.

### Impact of Ligand Aqueous Solubility

As an assay designed to determine *K*_d_ within the range of μM to low mM, the ligand concentration range was required to cover near mM concentration. We observed that for ligands with aqueous solubility below the desired nominal concentration, the R2KD assay results were impacted by the way samples were prepared. To illustrate this issue, an example is shown in Figure 7D. Using the soluble tool compound CCT365133, we prepared two data sets with different concentration series. For data set A, the ligand concentration was increasing from 50 to 250 μM (50, 100, 150, 200, and 250), then the concentration was kept at 300 μM for the last three data points to mimic the scenario, the compound has reached its aqueous solubility. For data set B, the ligand concentration continues to increase to 500 μM for the last three data points, mimicking the scenario that the compound has no solubility limit. The resulting *K*_d_ value for data set A was around 10-fold larger than data set B. So limited solubility portrays compounds as less active in R2KD assay. To prevent measured *K*_d_ values being impacted by this issue, we opted to prepare samples by making a series of dilutions of a ligand aqueous stock solution. Ligand aqueous solubility impacts all methods for *K*_d_ determination, the advantage of the NMR approach being that the actual concentration of ligand in samples could be monitored by extracting the first slice of the pseudo-2D R2 experiment where the relaxation delay is set to 4 ms. We have also been using quantitative ^1^H-NMR to measure the ligand concentration in the samples that contain no protein to further gauge the actual concentration of compound. If desired, the curve fitting could use the experimentally measured concentration range instead of nominal concentrations.

### Impact of Protein Concentration to *K*_d_ Value

Protein concentration was observed to be a key factor for the success of the R2KD assay. The concentration should be theoretically kept as low as possible to comply with the above derivations (i.e., L_T_/P_T_ ≫ 1) and ideally less than 20% of the lowest ligand concentration. If a tool compound is available, it is recommended a few concentrations be tested during assay optimization. However, we observed that *K*_d_ value is not dependent on the total protein concentration. Using compound CCT365133, *K*_d_ was determined using three different protein concentrations: 5, 25, and 35 μM. The results suggested that the variation was small (RSD 17%) (Figure 7E). At higher protein concentration (35 μM), we noticed that in samples with lower ligand concentration, such as 50 and 100 μM, *R*_2_ values had larger error bars between replicates. Two reasons are behind this increase in variability: first the NMR signals from protein became visible at 35 μM and if overlapped with ligand signals, the *R*_2_ values measured was a mixture from both; second, the *R*_2_ values of ligands became larger than 10 and could not be measured accurately using the *R*_2_ NMR experiment settings in the current method, as we discussed earlier in the *R*_2_ Measurement section. Protein concentrations higher than 35 μM were not used because the ligand *R*_2_ value could not be observed accurately with higher protein concentration for compound CCT365133. As expected, increased *R*_2_ values were observed with increased protein concentration due to higher percentages of bound ligand. As a rule of thumb, we suggest starting the assay development with a protein concentration of 5 μM. For large proteins (>50 kDa), the concentration may be further reduced to 2 μM while for small proteins (<30 kDa), the concentration can be increased to 10 μM.

### Curve Fitting Parameters

In theory, all NMR signals from the same molecule should share a single *K*_d_ value. So when fitting the *K*_d_ value using Graphpad Prism, a constraint “Shared value for all data sets” was used for *K*_d_ parameter fitting. For each shared parameter, Prism finds one (global) best-fit value that applies to all the data sets. This method also improved robustness of the fitting. The α values were not shared since they differ, depending on the individual proton’s environment. Once the α values were fitted, the bound protein concentration [PL] could be calculated using eqs 10 and 14 for individual NMR signals. This allowed us to simplify the graphical representation of the curve fitting (Figure 7F) and facilitated easier comparison of different compounds with a unified *y*-axis scale. Using such a visual representation, the graph would plateau at the total protein concentration used in the assay.

### Application

**Figure 8:**
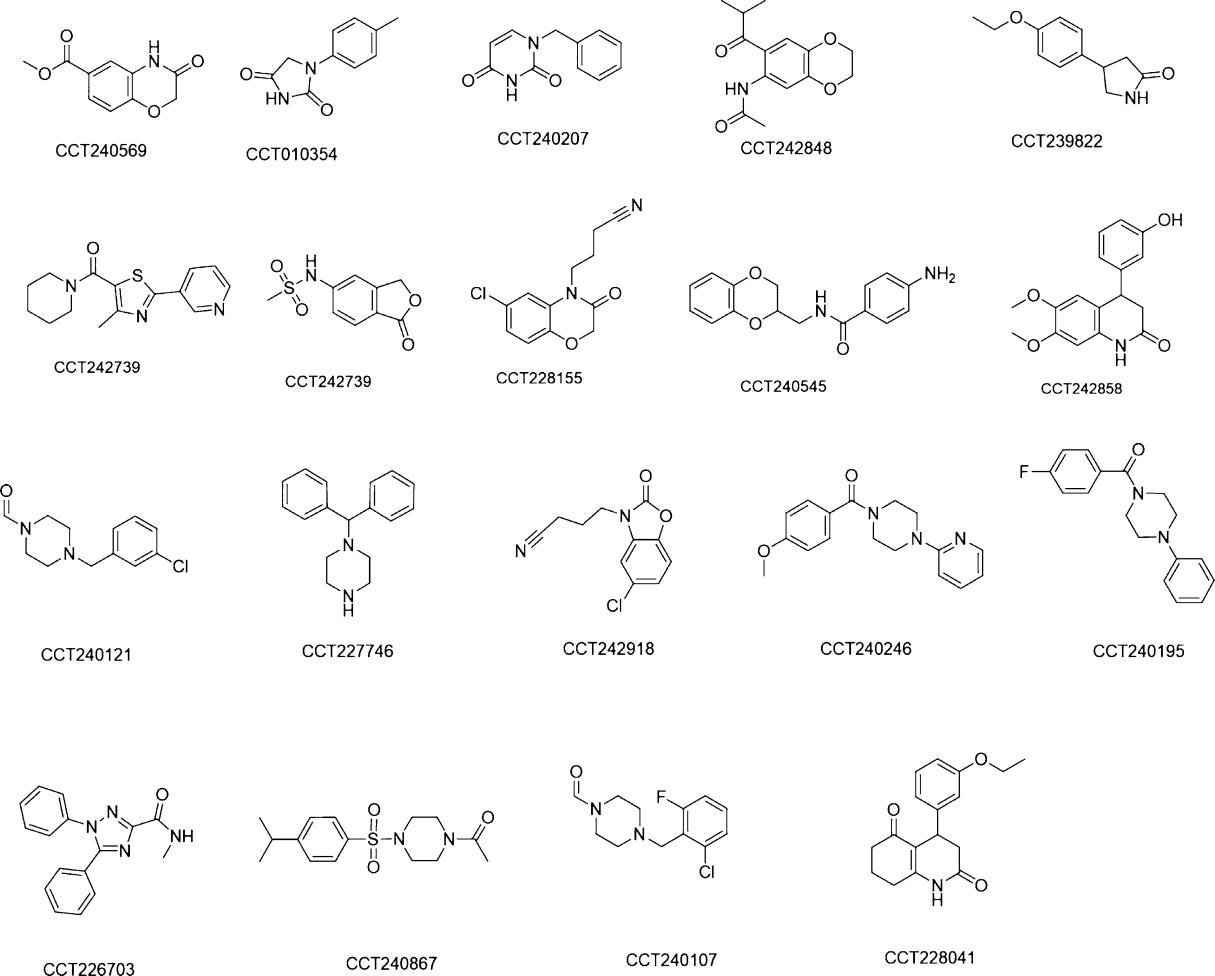
Structures of 19 fragment hits for the CRBN/DDB1 complex.

Here, we present how we used the R2KD assay to triage fragment hits against the CRBN/DDB1 complex. The CRBN/DDB1 complex is part of the E3 ligase system, with its thalidomide-binding domain (TBD) pocket critical for recruiting its substrate protein.^20^ A fragment library, composed of around 1000 compounds, was screened using *R*_2_ relaxation edited ^1^H-NMR experiments, with 19 fragments subsequently identified as competitive hits for the TBD pocket. The *R*_2_ values of these fragments were measured at two ligand concentrations (200 and 50 μM) against 2 μM of the CRBN/DDB1 complex. This enabled preliminary ranking of hits. The hits with larger *R*_2_ differences rank higher. This practice also reveals possible aggregators which have larger *R*_2_ values at higher concentration and will result in a non-saturating dose–response curve. We then measured the *K*_d_ values of the top 10 fragments using R2KD assay and confirmed the ranking order (Table 2). We also determined IC_50_ of the top 10 fragments using a FP based biochemical assay.^17^ Most fragments showed good correlation between the two assays apart from two weaker binders with IC_50_ > 3000 μM in FP assay (Table 2). The structures of these fragment hits are shown in Figure 8. Several of the most potent fragments contain similar cores such as uracil and hydantoin, as reported in a previous fragment screen.^21^

**Table 2:** *R*_2_ Values of the CRBN/DDB1 Complex Fragment Hits at Two Concentrations, *K*_d_ Values from R2KD Assay, and IC50 Values from Biochemical Assay

	average R_2_				
CCT	compound at 200 μM	compound at 50 μM	differences	R2KD assay *K*_d_ (μM)	biochemical assay IC_50_ (μM)
CCT240569	2.6	7.2	4.6	72	153
CCT010354	2	4.9	2.9	122	240
CCT240207	1.2	3.4	2.2	86	168
CCT242848	2.5	3.8	1.2	532	>3000
CCT239822	2.4	3.6	1.2	532	780
CCT242739	4.8	5.7	0.9	695	2501
CCT224736	1.6	2.4	0.9	729	1852
CCT228155	2.2	3	0.8	798	1643
CCT240545	1.5	2	0.5	638	>3000
CCT242858	2.1	2.5	0.5	1200	1365
CCT240121	3.3	3.8	0.4	ND^a^	ND
CCT227746	2.2	2.6	0.4	ND	ND
CCT242918	1.3	1.6	0.3	ND	ND
CCT240246	1.3	1.5	0.2	ND	ND
CCT240195	1.4	1.6	0.2	ND	ND
CCT226703	1.4	1.5	0.1	ND	ND
CCT240867	1.1	1.2	0.1	ND	ND
CCT240107	1.5	1.6	0.1	ND	ND
CCT228041	1.5	1.6	0.1	ND	ND

a ND: not determined.

## Conclusions

Here, we present a new, robust, versatile ligand-based NMR based approach to determine fragment binding *K*_d_ applicable to a range of targets. Based on the single site reversible binding theory and the Swift-Connick formula, we have devised a new equation to determine binding dissociation constant (*K*_d_) using transverse relaxation rate *R*_2_. We established an automated biophysical assay, R2KD, using state-of-the-art NMR instrumentation and optimized the approach for accuracy and reproducibility. Our results suggested good agreement of the *K*_d_ values from the R2KD assay with other biochemical and biophysical techniques across multiple protein targets with a range of molecular sizes. From our limit of detection study, we concluded that the R2KD assay is most suited to measure weak binding events in the *K*_d_ range of 10 μM to 1 mM. This suggests that the assay can be applied to triage hits resulting from a fragment-based drug discovery approach. We successfully demonstrated the use of this protocol to rank fragment hits from our NMR-based fragment screen against the CRBN/DDB1 complex of the CUL4^CRBN^ E3 ligase.

We envision that the R2KD assay will play a key role in fragment-based drug discovery, especially when allosteric sites are considered, for which other assays may not be readily available. It would be a valuable tool to assess primary hits from many forms of fragment screening techniques, such as crystallography or DSF screening. The R2KD assay can also serve as an orthogonal approach to biochemical assays in the early drug discovery stage when the initial hits are discovered and are in the 10–1000 μM *K*_d_ range.

## Experimental Section

### Materials

All compounds used in this study were either purchased from Chembridge or synthesized in house. All compounds where *K*_d_ are measured have purity higher than 95% by high-performance liquid chromatography (HPLC). NMR data were collected at 298 K on a Bruker AVANCE NEO 600 MHz spectrometer, equipped with 5 mm TCI CryoProbe using Bruker Topspin 4.0. HRMS data were collected using an Agilent 1200 series HPLC instrument and diode array detector coupled to a 6530 time-of-flight mass spectrometer with an ESI-AJS source. The characterization information is included in the Supporting Information, and all proteins used in this study were prepared in house, and the relevant information were published previously.

### *R*_2_ Determination

NMR data were collected at 298 K on a Bruker AVANCE NEO 600 MHz spectrometer, equipped with 5 mm TCI CryoProbe using Bruker Topspin 4.0. The *T*_2_ relaxation experiment was acquired using pulse program CPMG with 3-9-19 pulse sequence with gradients incorporated to suppress the water signal.^22^ Extra water suppression was achieved by adding presaturation during D1. The spin-echo period (delay-180°-delay) was set to 1 msec (d20 is 500 μs), and the relaxation delay (d1) was set to 10 s. The pseudo-2D experiment contained nine slices with spin-echo period repeated the following times: 4, 20, 100, 200, 300, 500, 700, 1000, and 2000. The *T*_2_ relaxation experiment was processed using MestReNova 14.1, and Data Analysis Module of MestReNova was used to obtain integrals of individual ^1^H-NMR signals, which were used to calculate *R*_2_ with equation *I* = *I*_0_exp (−*tR*_2_).

### Nonlinear Regression Analysis

Nonlinear regression analysis was carried out using GraphPad Prism 9.2.0. Equation 12 was defined in user-defined equations as: *Y* = 0.5α*((*X* + Kd + P) −sqrt((sqr(*X* + Kd + P)) – 4*P*X)) where *Y* was calculated from eq 13 and *X* is the total ligand concentration. Three parameters: P, α, and Kd were in the equation with the initial value set at 1. In the default constraints setting, P was “constant equal to” the total protein concentration, Kd was “shared value for all data sets”, and α was “must be greater than 10”. CIs of parameters was calculated at 95% level using Asymmetrical (Profile-likelihood) CI.

## Supporting Information Available

Detailed equation derivation; compounds characterization by ^1^H NMR and LCMS; *K*_d_ curve fitting data for the top 10 fragments identified in fragment screening campaign against the CRBN/DDB1 complex; and correlation plot between p*K*_d_ and p*K*_i_ using data presented in Table 1 (PDF)

## Abbreviations

CPMG: Carr–Purcell–Meiboom–Gill pulse sequence
CSP: chemical shift perturbation
DMSO: dimethyl sulfoxide
DSF: differential scanning fluorimetry
FBDD: fragment-based drug discovery
LONMR: ligand-observed NMR
NMR: nuclear magnetic resonance
NOE: nuclear Overhauser effect
R2KD: R2-based KD determination
SPR: surface plasmon resonance
STD: saturation transfer difference
