# Supplementary material for: Determination of Ligand-Binding Affinity (Kd) Using Transverse Relaxation Rate (R2) in the Ligand-Observed 1H NMR Experiment and Applications to Fragment-Based Drug Discovery
Source: J Med Chem. 2023 Jul 19;66(15):10617–27. doi: 10.1021/acs.jmedchem.3c00758 (PMC10424183; doi:10.1021/acs.jmedchem.3c00758)
Supplement: Supplementary file 1 — jm3c00758_si_001.pdf [file jm3c00758_si_001.pdf]

Supporting Information

Determination of Ligand Binding Affinity (K_d) using Transverse Relaxation Rate (R_2) in Ligand Observed ^{1}H NMR experiment and Applications to Fragment Based Drug Discovery

Manjuan Liu,[†,*] Amin Mirza,[†] P. Craig McAndrew,[†] Arjun Thapaliya,[†] Olivier A. Pierrat,[†] Mark Stubbs,[†] Tamas Hahner,[†] Nicola E. A. Chessum,[†] Paolo Innocenti,[†] John J. Caldwell,[†] Matthew D. Cheeseman,[†] Benjamin R. Bellenie,[†] Rob L. M. van Montfort,[†,#] Gary Newton,[†] Rosemary Burke,[†] Ian Collins,[†] Swen Hoelder.[†]

†Centre for Cancer Drug Discovery and #Division of Structural Biology, The Institute of Cancer Research, London SM2 5NG, U.K.

Corresponding author's email: Maggie.liu@icr.ac.uk

Contents

Detailed equation derivation....................S2

Compounds characterisation by ^{1}H NMR and LCMS....................S5

- CCT010354-7 ....S5
- CCT240569-6 ....S6
- CCT373101-2 ....S7
- CCT369304-1 ....S8
- CCT365133-1 ....S9
- CCT367090-1 ....S10
- CCT040036-6 ....S11
- CCT240207-4 ....S12
- CCT242848-4 ....S13
- CCT239822-4 ....S14
- CCT242739-4 ....S15
- CCT224736-5 ....S16
- CCT228155-5 ....S17
- CCT240545-4 ....S18
- CCT242858-4 ....S19

K_d curve fitting data for the top ten fragments identified in fragment screening campaign against CRBN/DDB1 complex....................S20

A correlation plot between pK_d and pK_i using data presented in Table 1. ....S23

Detailed equation derivation

$$K_d = \frac{[P][L]}{[PL]}$$	(1)
$$[P] = P_T - [PL]$$	(2)
$$[L] = L_T - [PL]$$	(3)
$$K_d = \frac{(P_T - [PL])(L_T - [PL])}{[PL]}$$	(4)
$$K_d[PL] = P_T(L_T - [PL]) - [PL](L_T - [PL])$$	
$$P_T L_T - P_T[PL] - L_T[PL] + [PL]^2 - K_d[PL] = 0$$	
$$[PL]^2 - (P_T + L_T + K_d)[PL] + P_T L_T = 0$$	(5)
this is a quadratic equation<br>$ax^2+bx+c=0$<br>its solution is<br>$x = \frac{-b \pm \sqrt{b^2 - 4ac}}{2a}$<br>$x_1 = \frac{-b-\sqrt{b^2-4ac}}{2a}$ is chosen because [PL] must be less than $P_T or\ L_T$.<br>Here a = 1, b = $P_T + L_T + K_d$, c = $P_T L_T$<br>So the solution is:	
$$[PL] = \frac{1}{2}(P_T + L_T + K_d) - \frac{1}{2}\sqrt{(P_T + L_T + K_d)^2 - 4P_T L_T}$$	(6)
For example, for a system with<br>K_d 50 µM, P_T 25 µM, L_T 10 µM<br>That is a=1, b=-85, c=250<br>$F(x)=x^2-85x+250$ The two solution is x_1=3.05, x_2= 81.9<br>[PL] should be 3.05	

$$R_{2,obs} = \rho_F R_{2F} + \rho_F \rho_B K_{ex} \frac{\{R_{2B}(R_{2B} + \rho_F K_{ex}) + (\Delta\Omega)^2\}}{\{(R_{2B} + \rho_F K_{ex})^2 + (\Delta\Omega)^2\}}$$	(7)
Equation (7) can be further simplified to equation (8) as in the limit of fast exchange on the chemical shift and relaxation time scales, one observes only a single signal with an averaged relaxation rate $R_{2,obs}$	
$$R_{2,obs} = \rho_F R_{2F} + \rho_B R_{2B} + \rho_F \rho_B \frac{(\Delta\Omega)^2}{K_{ex}}$$	(8)
$$R_{2,obs} = (1 - \rho_B) R_{2F} + \rho_B R_{2B} + (1 - \rho_B)\rho_B \frac{(\Delta\Omega)^2}{K_{ex}}$$	
$$R_{2,obs} = R_{2F} + \left(R_{2B} - R_{2F} + \frac{(\Delta\Omega)^2}{K_{ex}}\right)\rho_B - \frac{(\Delta\Omega)^2}{K_{ex}} {\rho_B}^2$$	
$$R_{2,obs} = R_{2F} + \left(R_{2B} - R_{2F} + \frac{(\Delta\Omega)^2}{K_{ex}}\right)\frac{[PL]}{L_T} - \frac{(\Delta\Omega)^2}{K_{ex}}\left(\frac{[PL]}{L_T}\right)^2$$	(9)
$$R_{2,obs} = R_{2F} + \left((R_{2B} - R_{2F})\frac{[PL]}{L_T} + \frac{(\Delta\Omega)^2}{K_{ex}}\left(\frac{[PL]}{L_T} - \left(\frac{[PL]}{L_T}\right)^2\right)\right)$$	
Assuming using 10 fold excess of ligand, in the weak binding range (1-1000 µM), ρ_B is less than 0.1, as a result, ${\rho_B}^2$ is 10 fold less than ρ_B, so the term $\frac{(\Delta\Omega)^2}{K_{ex}}(\frac{[PL]}{L_T})^2$ in equation (9) can be ignored	
$$R_{2,obs} = R_{2F} + \left(\left(R_{2B} - R_{2F} + \frac{(\Delta\Omega)^2}{K_{ex}}\right)\left(\frac{[PL]}{L_T}\right)\right)$$	
$$R_{2,obs} - R_{2F} = \left(\left(R_{2B} - R_{2F} + \frac{(\Delta\Omega)^2}{K_{ex}}\right)\left(\frac{[PL]}{L_T}\right)\right)$$	
$$[PL] = \frac{R_{2,obs} - R_{2F}}{R_{2B} - R_{2F+}\frac{(\Delta\Omega)^2}{K_{ex}}} L_T$$	(10)
As from previous section we know: $[PL] = \frac{1}{2}(P_T + L_T + K_d) - \frac{1}{2}\sqrt{(P_T + L_T + K_d)^2 - 4P_T L_T}$	(6)
$$(R_{2,obs} - R_{2F})L_T = \frac{1}{2}(R_{2B} - R_{2F} + \frac{(\Delta\Omega)^2}{K_{ex}})\left\{(P_T + L_T + K_d) - \sqrt{(P_T + L_T + K_d)^2 - 4P_T L_T}\right\}$$	(11)
$$y = \frac{1}{2}\alpha\left\{(P_T + L_T + K_d) - \sqrt{(P_T + L_T + K_d)^2 - 4P_T L_T}\right\}$$	(12)

$$y = (R_{2,obs} - R_{2F})L_T$$	(13)
$$\alpha = \left(R_{2B} - R_{2F} + \frac{(\Delta\Omega)^2}{K_{ex}}\right)$$	(14)

A few words about α

α is so called amplification factor which enable NMR to detect weak interactions when the bound fraction of ligand could be lower than 1%.

For different ^{1}H-NMR signals, α varies because they reside in the different environments when interact with protein.

Three factors will influence α values: R_{2B}, ΔΩ, and K_{ex}

R_{2B} is R_2 relaxation rate when ligand is bound to protein and for ^{1}H nucleus, the transverse relaxation occurs mainly through dipole-dipole interaction and can be expressed with equation 15

$$R_{2,DD} = \frac{(\mu_0/4\pi)^2\hbar^2\gamma_H^4}{8}\sum_{j=1}^{N}\frac{1}{r_j^6}\left(5\tau_c + \frac{9\tau_c}{1+\omega_H^2\tau_c^2} + \frac{6\tau_c}{1+4\omega_H^2\tau_c^2}\right) \quad (15)$$

$R_{2,DD}$ is the R_2 through dipole dipole interaction

$\hbar$ is the Planck constant divided by 2π (1.055 x 10^{-34} J S)

$\gamma_H$is gyromanetic ratio of nucleus ($T^{-1} s^{-1}$)

μ_0 is vacuum permeability (4π x 10^{-7} H m^{-1})

r: distance between two nuclei (m)

τ_c is the rotational correlation time (s)

ω_H is nutationa frequency of nucleus (s^{-1})

So the value of R_{2B} is proportional to the number of nearby protein protons which can form dipolar-dipolar interaction with ligand proton. As a result, ligand moiety inside the binding pocket is more likely to show higher R_{2B}. Another contributing factor to the α value is $\frac{(\Delta\Omega)^2}{K_{ex}}$. while ΔΩ (chemical shift difference) depends on the nature of the nearby protein atoms to the ligand proton, for example a benzene ring or the hydrogen bond produces a larger chemical shift difference than a methyl group. The impact of ΔΩ depends on the exchange rate K_{ex}, which lies in the range of 1000 to 100000 $S^{-1}$for a weakly binding compound with $K_d \geq 100$ µM. So with the right combination of ΔΩ and K_{ex}, this factor could contribute to α value in a meaningful way. In summary, the different α values rising from different ^{1}H NMR signals of the ligand could be an indicator that ligand bound in a specific orientation. However, due to the multiple factors affecting the α value, its accuracy as indicators of binding epitope requires case by case study.

Compounds characterisation by ^{1}H NMR and LCMS

CCT010354-7

1H NMR (600 MHz, DMSO) δ 11.13 (s, 1H), 7.50 – 7.45 (m, 2H), 7.18 (d, J = 8.3 Hz, 2H), 4.41 (s, 2H), 2.27 (s, 3H). HRMS (ESI+): calcd for $C_{10}H_{10}N_2O_2$ $(M + H)^+$, 191.0815; found 191.0817.

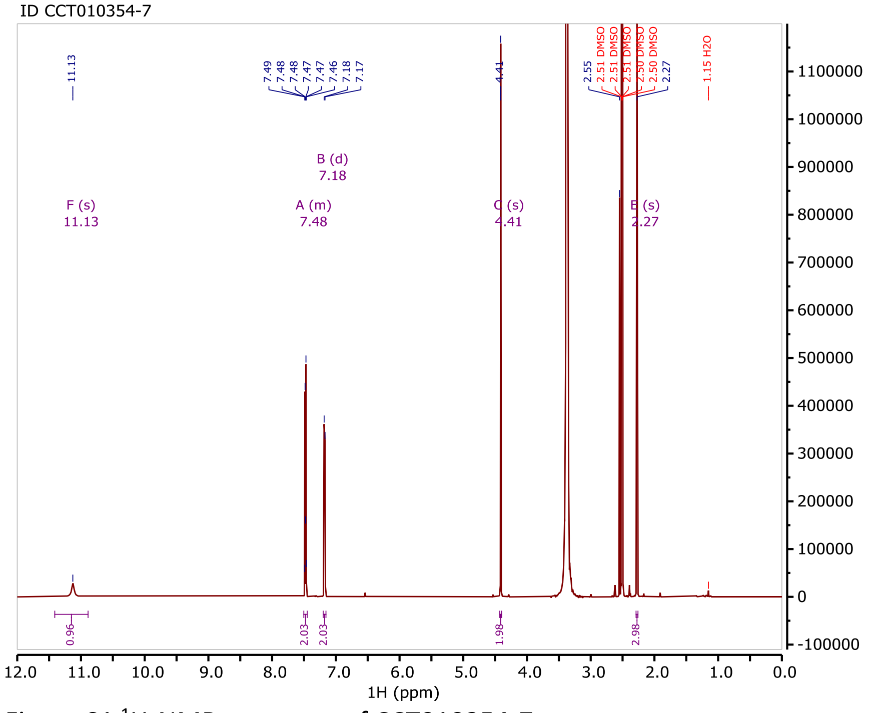

Parameter	Value
Title	ML-5245-007-CCT010354-7.10.fid
Temperature	298.0
Pulse Sequence	zg
Probe	Z75812_0002 (CP TCI 600S3 H-C/ N-D-05 Z)
Number of Scans	32
Receiver Gain	50.3
Relaxation Delay	20.0000
Acquisition Date	2022-03-18T10:22:20
Spectrometer Frequency	600.13

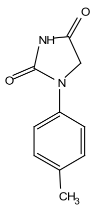

Figure S1 ^{1}H-NMR spectrum of CCT010354-7

	Parameter	Value
1	Data Path	D:\ MassHunter\ Walkup\ DataFiles\ mliu\ QT22_02121_007.d
2	Acquired Date	2022-03-23T12:58:33.296Z
3	Sample Name	QT22_02121
4	Comment	ML-010354
5	Sample Position	P1-A2

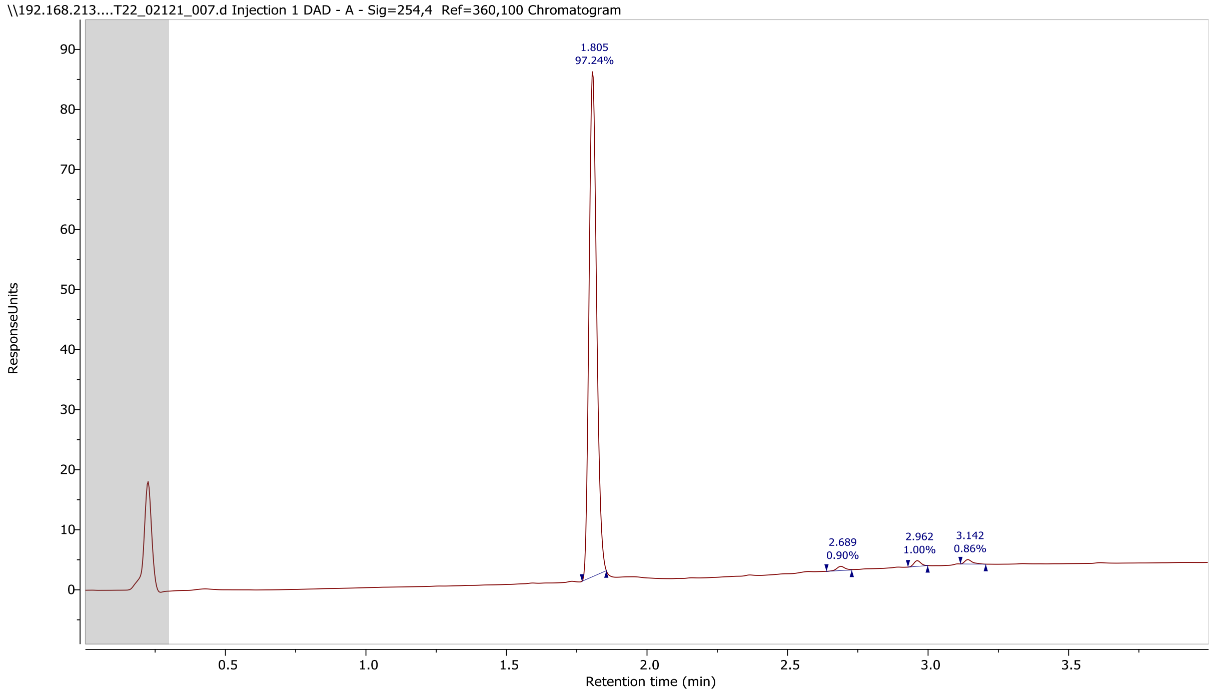

Figure S2 LC-UV trace of CCT010354-7

CCT240569-6

1H NMR (600 MHz, DMSO) δ 10.89 (s, 1H), 7.55 (dd, J = 8.4, 2.0 Hz, 1H), 7.52 (d, J = 2.0 Hz, 1H), 7.05 (d, J = 8.4 Hz, 1H), 4.69 (s, 2H), 3.82 (s, 3H). HRMS (ESI+): calcd for C10H9NO4 (M + H)+, 208.0604; found 208.0601.

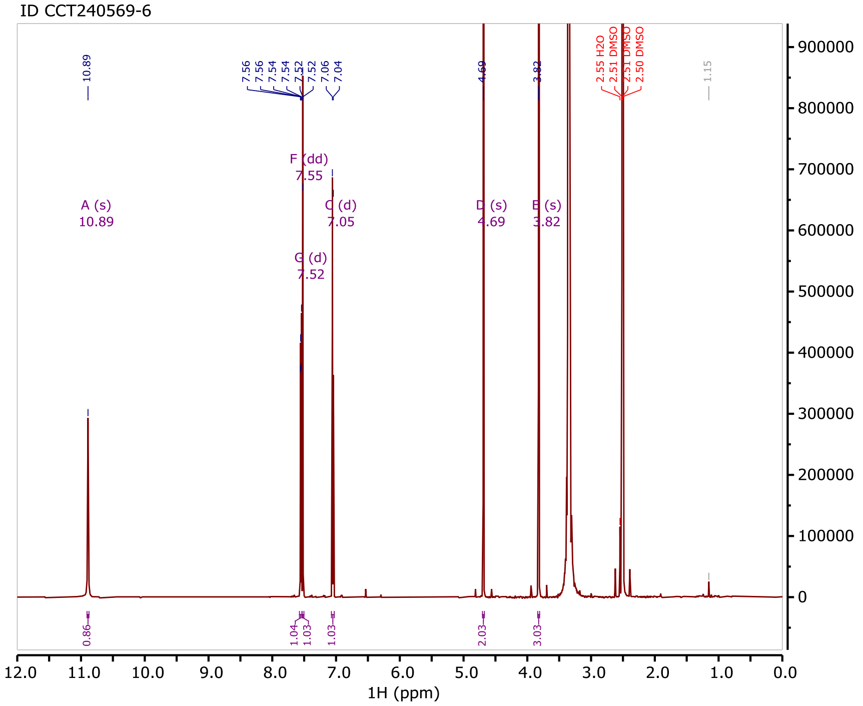

Parameter	Value
Title	ML-5245-007-CCT240569-6.10.fid
Temperature	298.0
Pulse Sequence	zg
Probe	Z75812_0002 (CP TCI 600S3 H-C/ N-D-05 Z)
Number of Scans	32
Receiver Gain	101.0
Relaxation Delay	20.0000
Acquisition Date	2022-03-18T11:10:08
Spectrometer Frequency	600.13

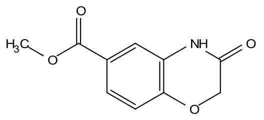

Figure S3 ^{1}H-NMR spectrum of CCT240569-6

	Parameter	Value
1	Data Path	D:\ MassHunter\ Walkup\ DataFiles\ mliu\ QT22_02122_009.d
2	Acquired Date	2022-03-23T13:09:18.363Z
3	Sample Name	QT22_02122
4	Comment	ML-240569
5	Sample Position	P3-A1

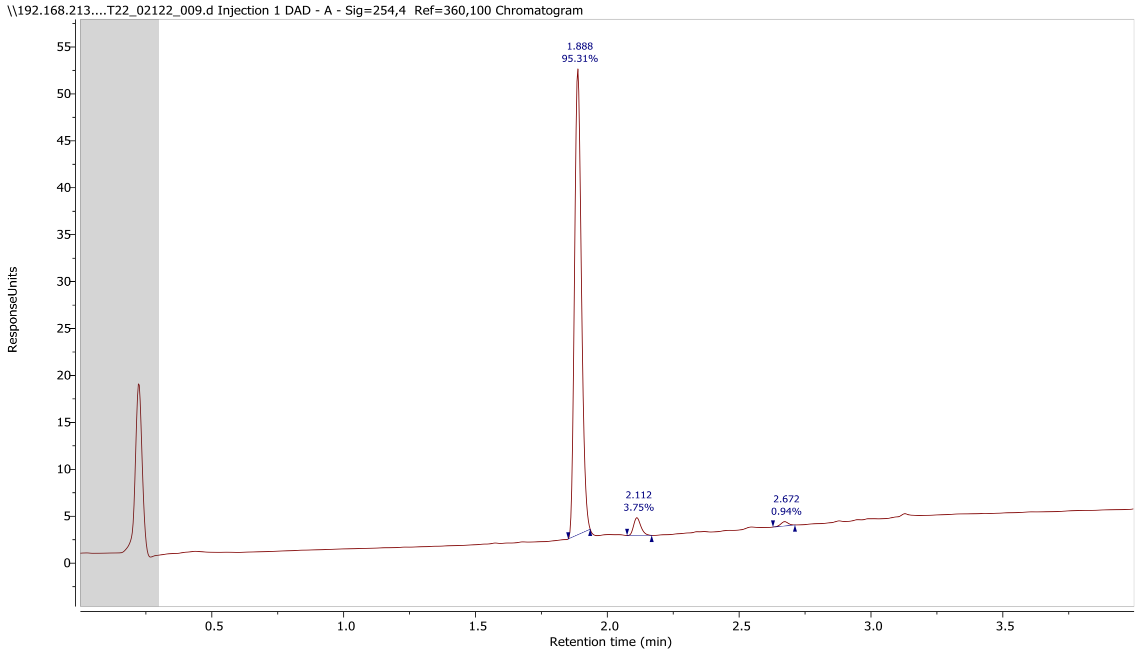

Figure S4 LC-UV trace of CCT240569-6

CCT373101-2

1H NMR (600 MHz, DMSO) δ 10.37 (s, 1H), 7.40 (t, J = 7.8, 7.8 Hz, 2H), 7.34 (d, J = 7.4 Hz, 2H), 7.24 (t, J = 7.3, 7.3 Hz, 1H), 3.80 (t, J 6.7 Hz, 2H), 2.71 (t, J = 6.7, 6.7 Hz, 2H).

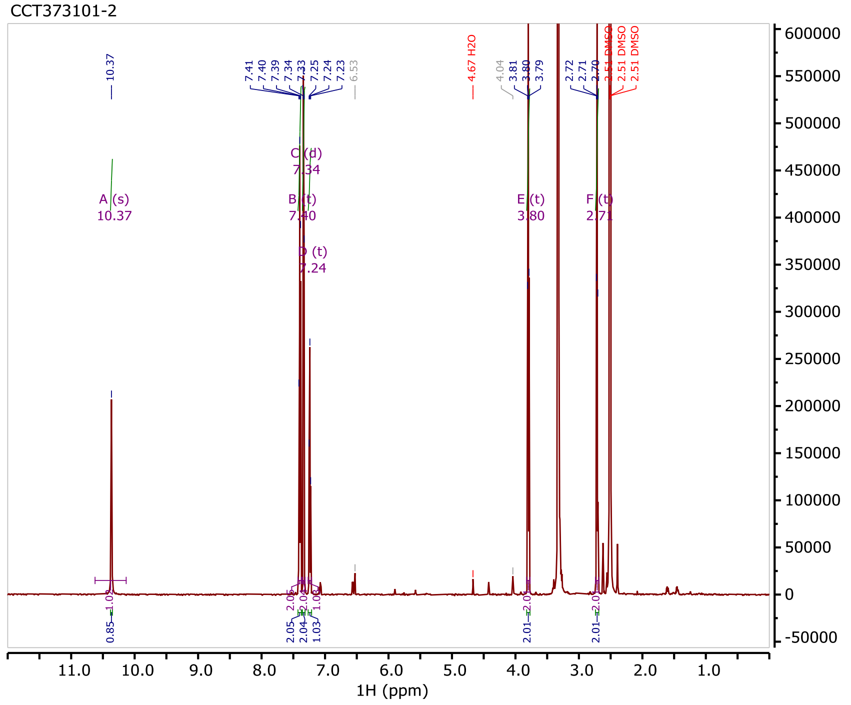

Parameter	Value
Title	ML-5245-038sto-CCT373101-2.10.fid
Temperature	298.0
Pulse Sequence	zg
Probe	Z75812_0002 (CP TCI 600S3 H-C/ N-D-05 Z)
Number of Scans	32
Receiver Gain	101.0
Relaxation Delay	20.0000
Acquisition Date	2021-05-11T18:07:32
Spectrometer Frequency	600.13

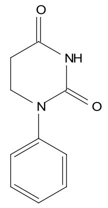

Figure S5 ^{1}H-NMR spectrum of CCT373101-2

	Parameter	Value
1	Data Path	D:\ MassHunter\ Walkup\ DataFiles\ mliu\ QT22_02123_012.d
2	Acquired Date	2022-03-23T13:25:28.742Z
3	Sample Name	QT22_02123
4	Comment	ML-373101
5	Sample Position	P3-A2

\\192.168.213....T22_02123_012.d Injection 1 DAD - A - Sig=254,4 Ref=360,100 Chromatogram

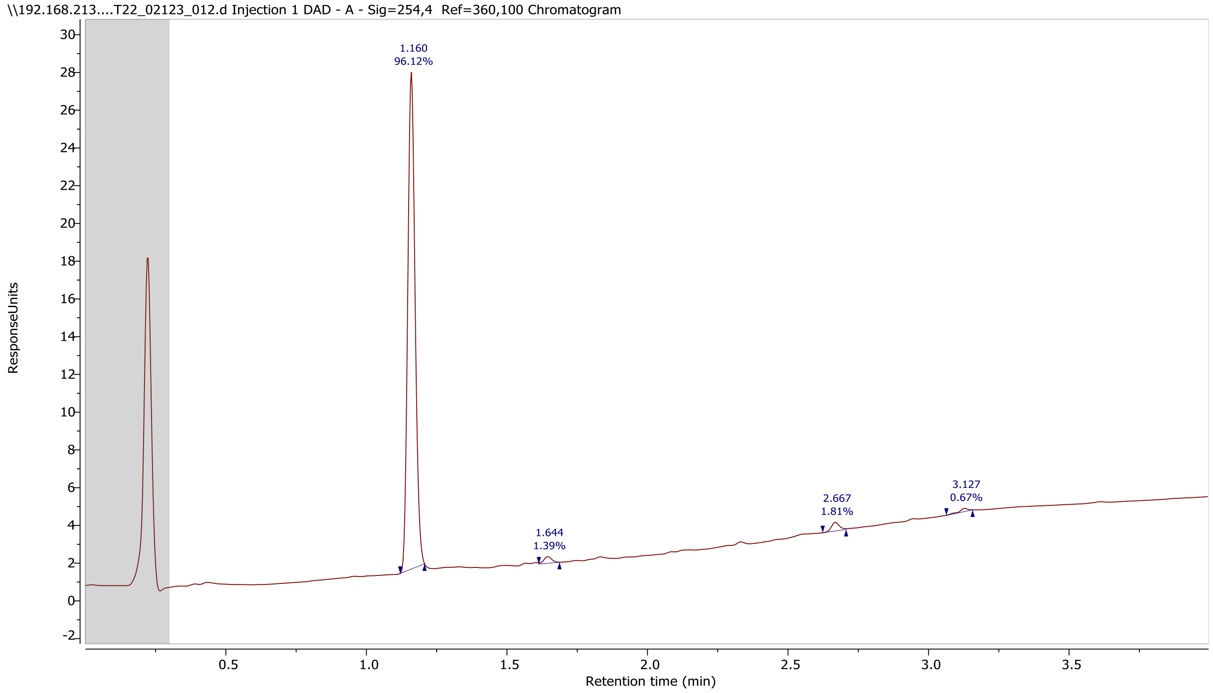

Figure S6 LC-UV trace of CCT373101-2

CCT369304-1

1H NMR (600 MHz, DMSO) δ 8.96 (s, 1H), 8.31 (t, J = 1.6, 1.6 Hz, 1H), 8.14 (dt, J = 7.8, 1.3, 1.3 Hz, 1H), 7.98 (dt, J = 7.9, 1.2, 1.2 Hz, 1H), 7.68 (t, J = 7.8, 7.8 Hz, 1H), 7.30 (dd, J = 7.7, 1.7 Hz, 1H), 7.14 – 7.10 (m, 1H), 7.10 – 7.03 (m, 2H), 2.46 – 2.41 (m, 4H), 1.53 (p, J = 5.9, 5.9, 5.6, 5.6 Hz, 4H), 1.46 – 1.40 (m, 2H). HRMS (ESI+): calcd for $C_{18}H_{20}N_2O_4S$ $(M + H)^+$, 362.1247; found 362.1231.

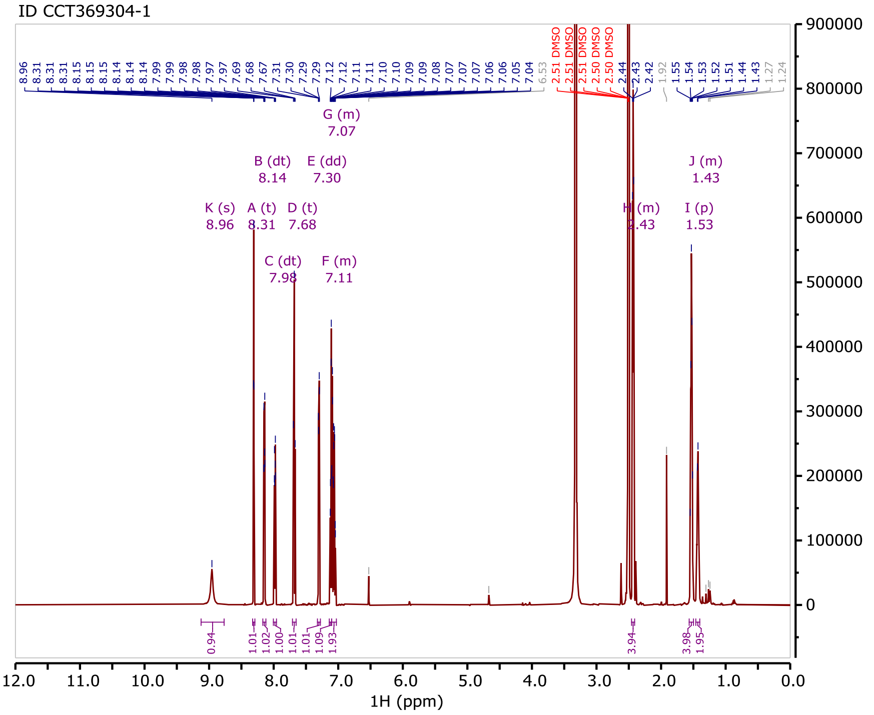

Parameter	Value
Title	ML-5245-096-CCT369304-1.10.fid
Temperature	298.0
Pulse Sequence	zg
Probe	Z75812_0002 (CP TCI 600S3 H-C/ N-D-05 Z)
Number of Scans	32
Receiver Gain	101.0
Relaxation Delay	20.0000
Acquisition Date	2021-11-09T11:43:56
Spectrometer Frequency	600.13

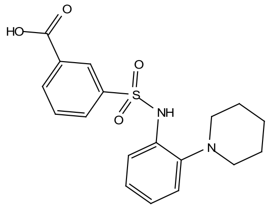

Figure S7 ^{1}H-NMR spectrum of CCT369304-1

	Parameter	Value
1	Data Path	D:\ MassHunter\ Walkup\ DataFiles\ mliu\ QT22_02124_013.d
2	Acquired Date	2022-03-23T13:30:48.440Z
3	Sample Name	QT22_02124
4	Comment	ML-369304
5	Sample Position	P3-A3

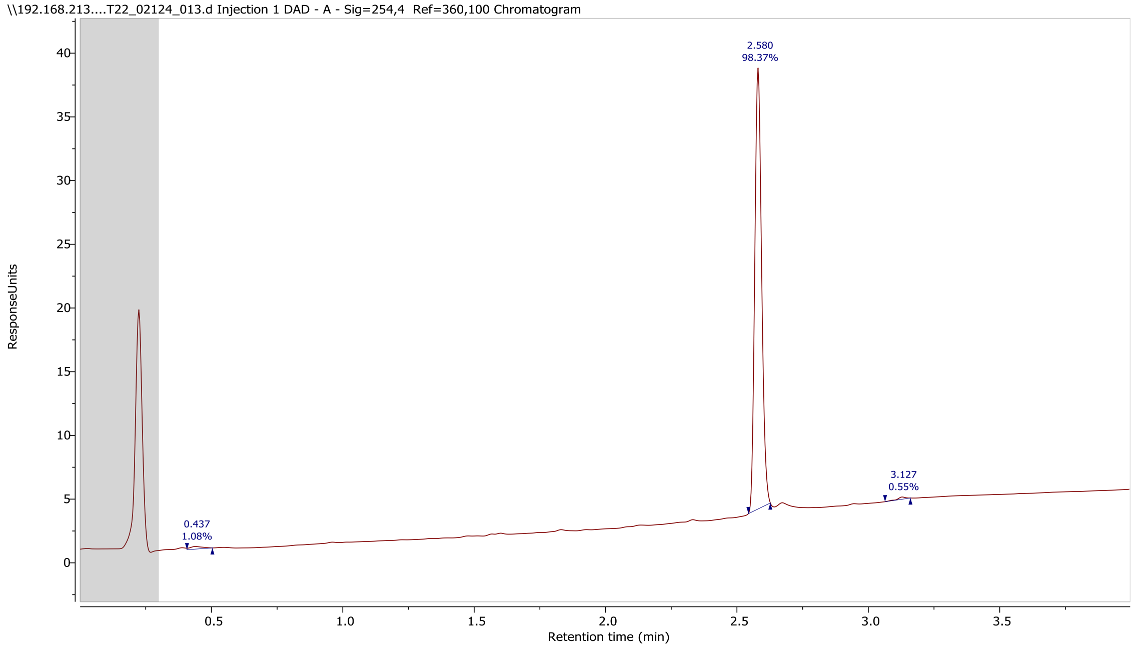

Figure S8 LC-UV trace of CCT369304-1

CCT365133-1

1H NMR (600 MHz, DMSO) δ 9.08 (s, 1H), 8.06 – 8.02 (m, 1H), 7.84 (s, 1H), 7.49 (s, 1H), 6.76 (d, J = 6.2 Hz, 1H), 4.14 (t, J = 5.6, 5.6 Hz, 2H), 3.75 (t, J = 5.6, 5.6 Hz, 2H). HRMS (ESI+): calcd for C11H10ClN5O (M + H)+, 264.0647; found 264.0633.

ID CCT365133-1

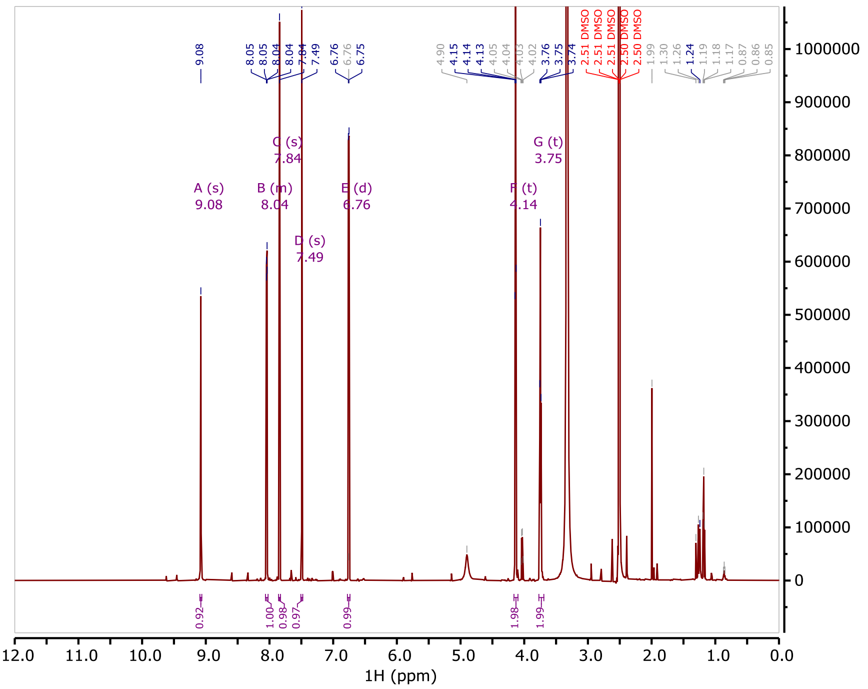

Parameter	Value
Title	ML-5245-053-CCT365133-1.10.fid
Comment	ID CCT365133-1
Probe	Z75812_0002 (CP TCI 600S3 H-C/ N-D-05 Z)
Number of Scans	32
Receiver Gain	101.0
Relaxation Delay	20.0000
Acquisition Date	2021-08-09T12:48:27
Spectrometer Frequency	600.13

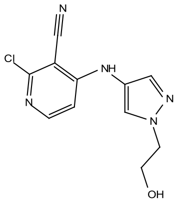

Figure S9 ^{1}H-NMR spectrum of CCT365133-1

	Parameter	Value
1	Data Path	D:\ MassHunter\ Walkup\ DataFiles\ mliu\ QT22_02128_021.d
2	Acquired Date	2022-03-23T14:26:30.895Z
3	Sample Name	QT22_02128
4	Comment	ML-365133
5	Sample Position	P1-A5

\\192.168.213....T22_02128_021.d Injection 1 DAD - A - Sig=254,4 Ref=360,100 Chromatogram

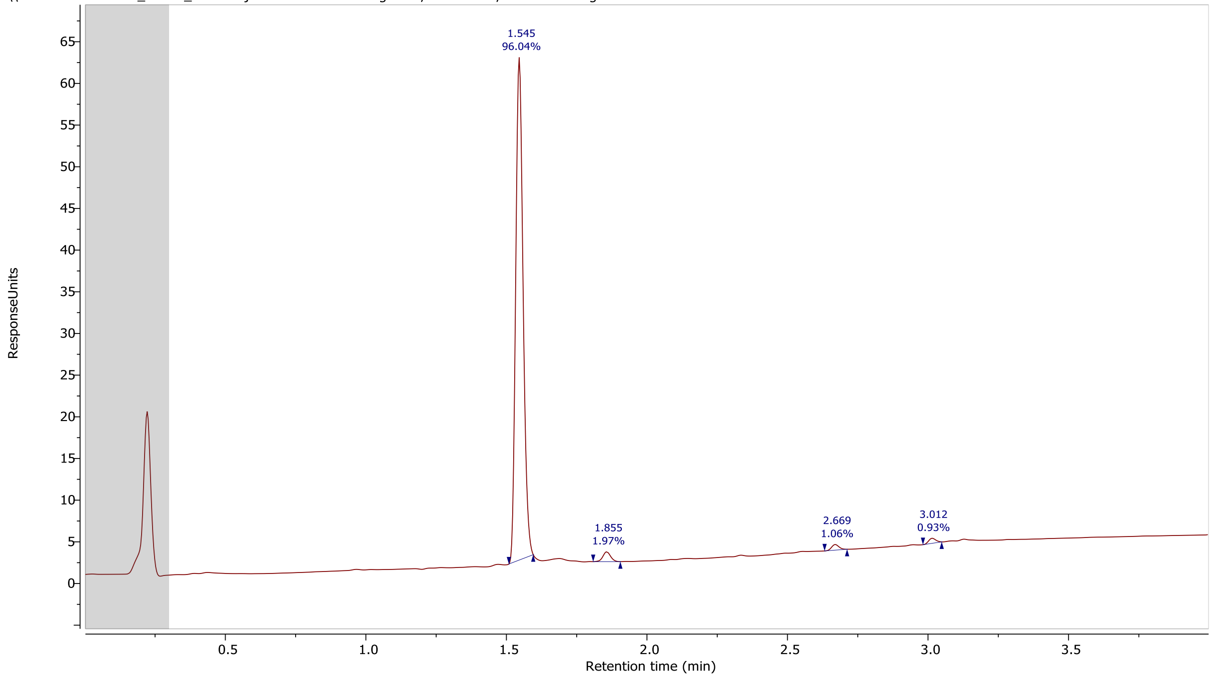

Figure S10 LC-UV trace of CCT365133-1

CCT367090-1

1H NMR (600 MHz, DMSO) δ 9.49 (s, 1H), 8.00 (d, J = 6.2 Hz, 1H), 7.19 (dd, J = 4.9, 3.2 Hz, 2H), 6.98 (dd, J = 8.3, 1.7 Hz, 1H), 6.67 (d, J = 6.2 Hz, 1H), 4.81 (d, J = 5.3 Hz, 1H), 3.79 – 3.64 (m, 3H), 3.36 (s, 3H), 1.45 (dtd, J = 14.6, 7.3, 7.3, 4.0 Hz, 1H), 1.33 (dp, J = 14.7, 7.3, 7.3, 7.3, 7.3 Hz, 1H), 0.91 (t, J = 7.4, 7.4 Hz, 3H). HRMS (ESI+): calcd for C18H18ClN5O2 (M + H)+, 372.1221; found 372.1213.

CCT367090-1

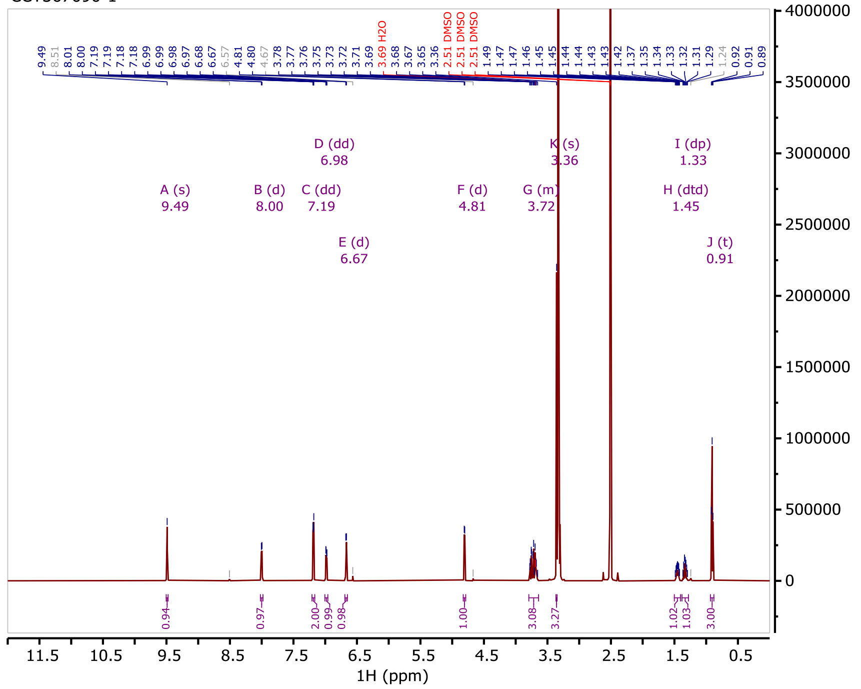

Parameter	Value
Title	ML-5245-039sto-CCT367090-1.10.fid
Comment	CCT367090-1
Probe	Z75812_0002 (CP TCI 600S3 H-C/ N-D-05 Z)
Number of Scans	32
Receiver Gain	101.0
Relaxation Delay	20.0000
Acquisition Date	2021-05-11T19:14:31
Spectrometer Frequency	600.13

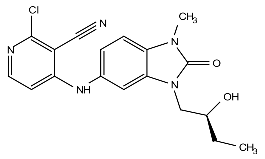

Figure S11 ^{1}H-NMR spectrum of CCT367090-1

	Parameter	Value
1	Data Path	D:\ MassHunter\ Walkup\ DataFiles\ mliu\ QT22_02127_022.d
2	Acquired Date	2022-03-23T14:32:16.513Z
3	Sample Name	QT22_02127
4	Comment	ML-367090
5	Sample Position	P3-A5

\\192.168.213....T22_02127_022.d Injection 1 DAD - A - Sig=254,4 Ref=360,100 Chromatogram

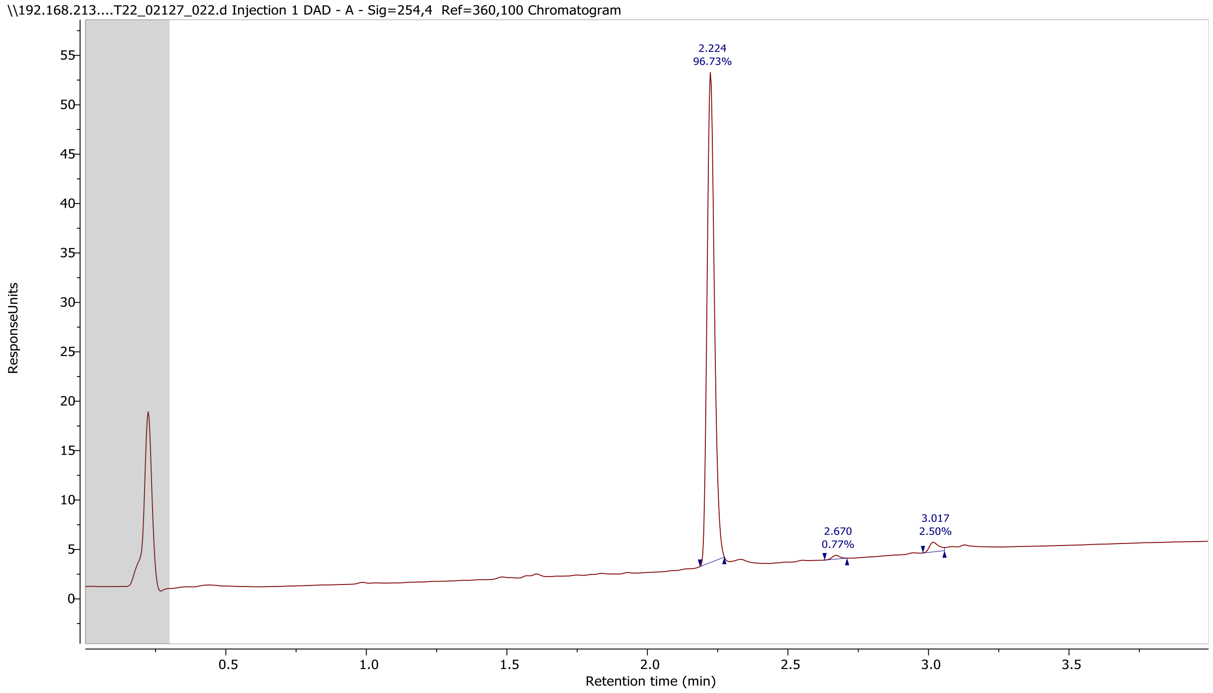

Figure S12 LC-UV trace of CCT367090-1

CCT040036-6

1H NMR (600 MHz, DMSO) δ 10.17 (s, 1H), 8.50 (s, 1H), 7.49 (ddt, J = 8.5, 5.7, 2.9, 2.9 Hz, 2H), 7.36 – 7.29 (m, 2H), 6.30 (s, 1H), 2.41 (s, 3H). HRMS (ESI+): calcd for C12H10FN5 (M + H)+, 244.0993; found 244.0986.

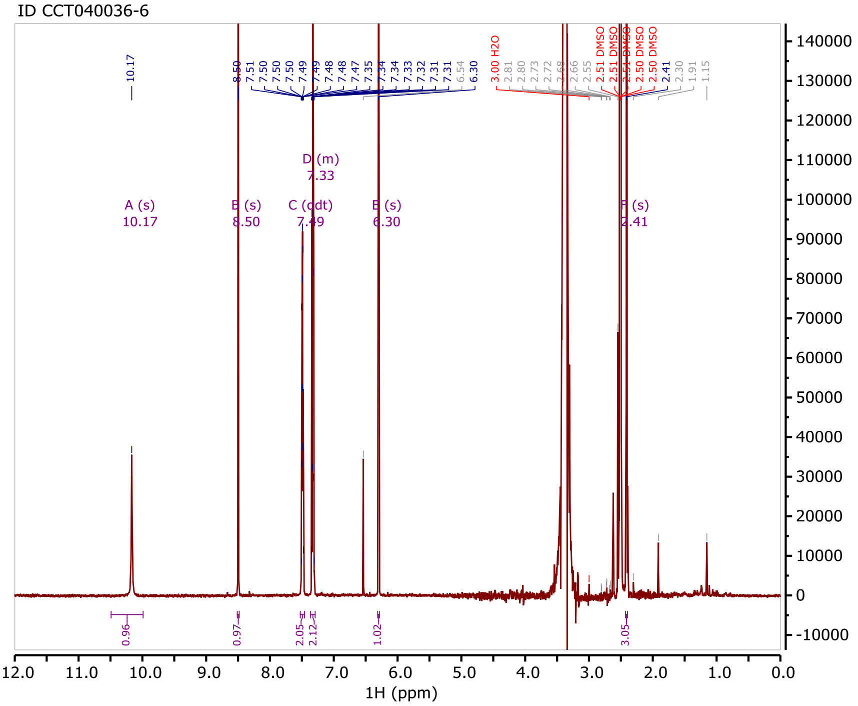

Parameter	Value
Title	ML-5245-007-CCT040036-6.10.fid
Temperature	298.0
Pulse Sequence	zg
Probe	Z75812_0002 (CP TCI 600S3 H-C/ N-D-05 Z)
Number of Scans	32
Receiver Gain	50.3
Relaxation Delay	20.0000
Acquisition Date	2022-03-18T10:46:58
Spectrometer Frequency	600.13

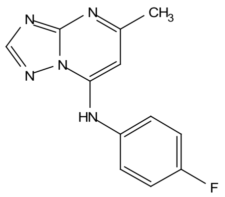

Figure S13 ^{1}H-NMR spectrum of CCT040036-6

	Parameter	Value
1	Data Path	D:\ MassHunter\ Walkup\ DataFiles\ mliu\ QT22_02129_025.d
2	Acquired Date	2022-03-23T14:48:25.283Z
3	Sample Name	QT22_02129
4	Comment	ML-040036
5	Sample Position	P3-A6

\\192.168.213....T22_02129_025.d Injection 1 DAD - A - Sig=254,4 Ref=360,100 Chromatogram

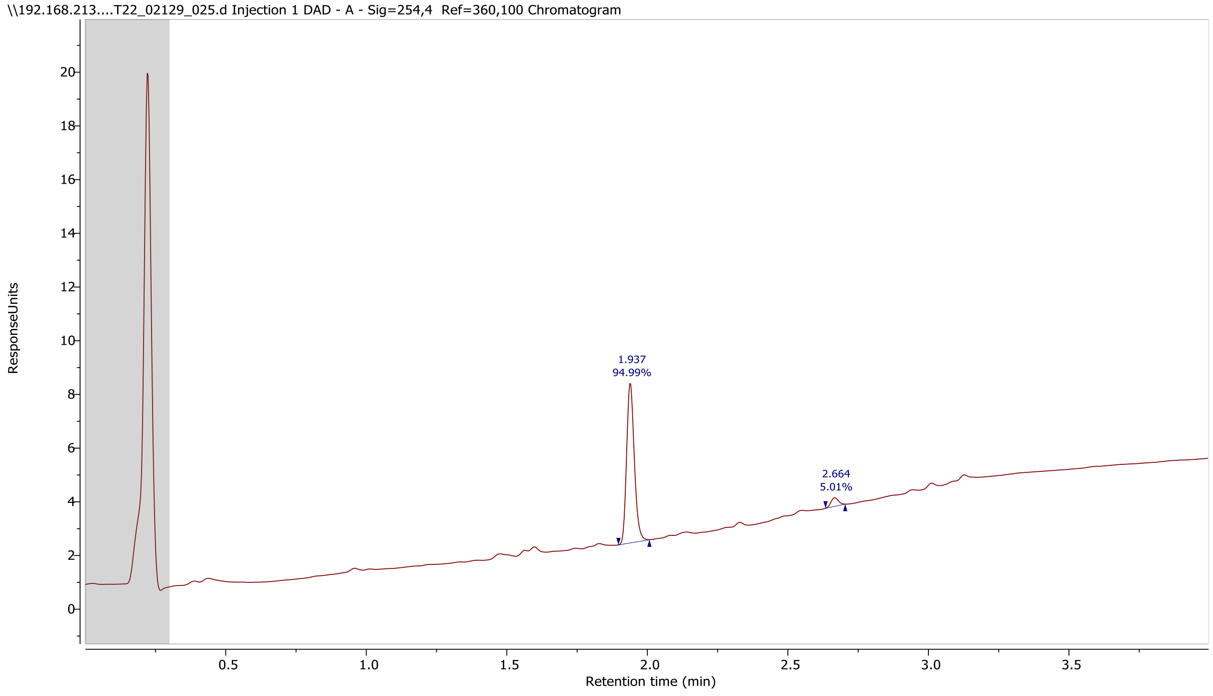

Figure S14 LC-UV trace of CCT040036-6

CCT240207-4

1H NMR (600 MHz, DMSO) δ 11.32 (s, 1H), 7.76 (d, J = 7.9 Hz, 1H), 7.37 (t, J = 7.4, 7.4 Hz, 2H), 7.34 – 7.27 (m, 3H), 5.60 (dd, J = 7.8, 2.2 Hz, 1H), 4.88 (s, 2H). HRMS (ESI+): calcd for $C_{11}H_{10}N_2O_2$ (M + H)+, 203.0815; found 203.0799.

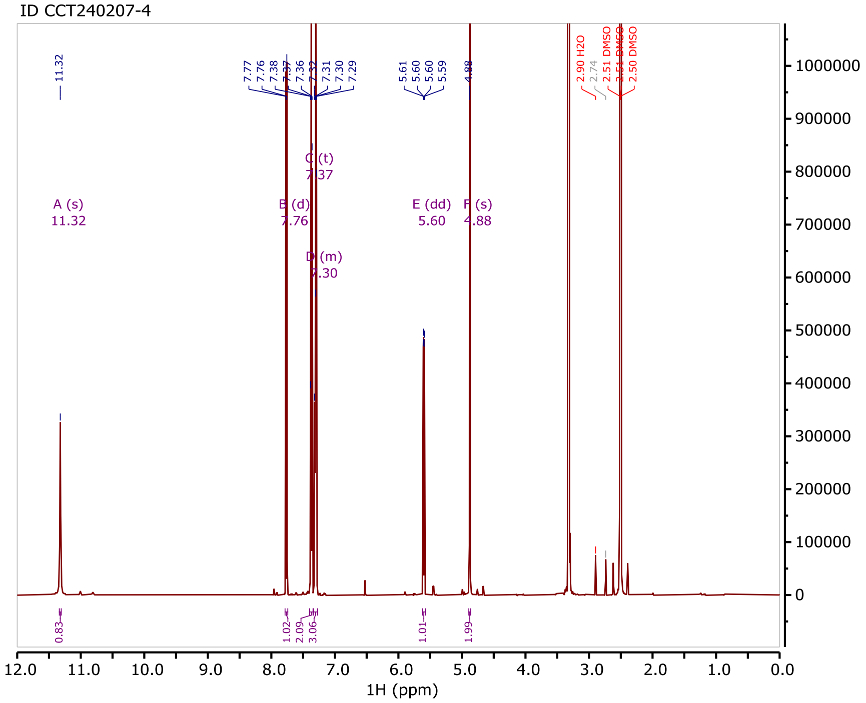

Parameter	Value
Title	ML-5245-069-CCT240207-4.10.fid
Comment	ID CCT240207-4
Probe	Z75812_0002 (CP TCI 600S3 H-C/ N-D-05 Z)
Number of Scans	32
Receiver Gain	101.0
Relaxation Delay	20.0000
Acquisition Date	2021-09-05T04:09:16
Spectrometer Frequency	600.13

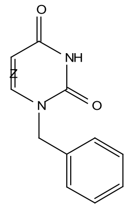

Figure S15 ^{1}H-NMR spectrum of CCT240207-4

	Parameter	Value
1	Data Path	D:\ MassHunter\ Walkup\ DataFiles\ mliu\ QT22_02130_027.d
2	Acquired Date	2022-03-23T14:59:20.364Z
3	Sample Name	QT22_02130
4	Comment	ML-240207
5	Sample Position	P1-A6

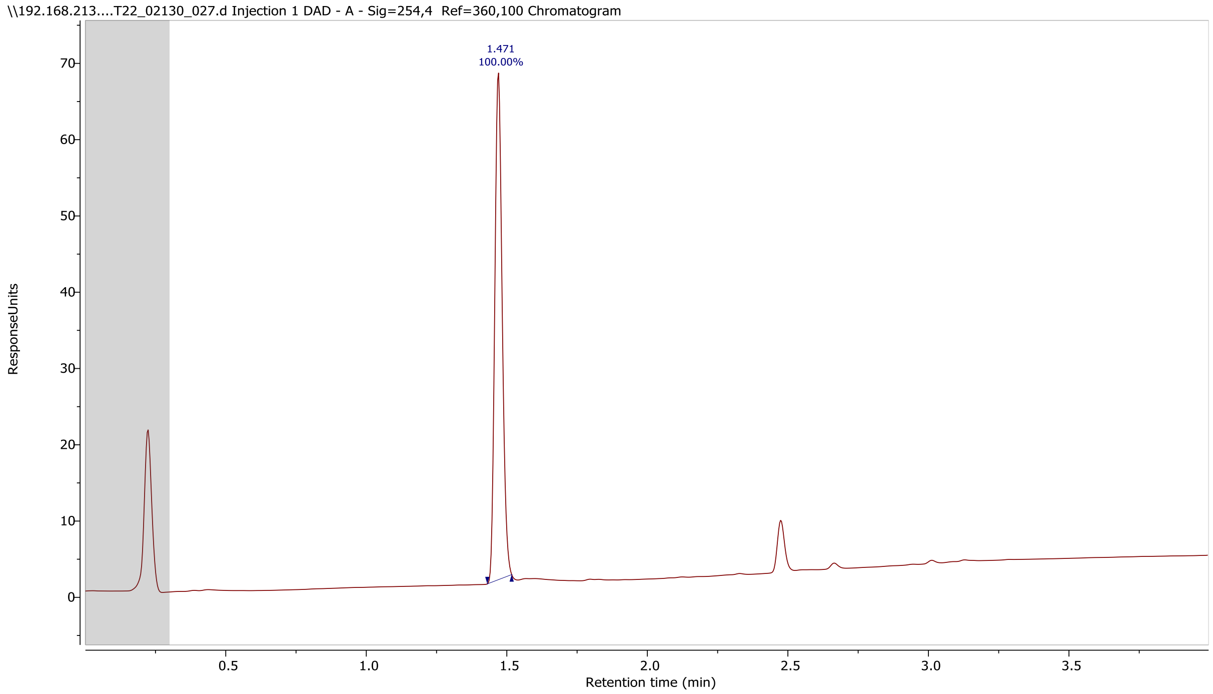

Figure S16 LC-UV trace of CCT240207-4

CCT242848-4

1H NMR (600 MHz, DMSO) δ 11.38 (s, 1H), 8.00 (s, 1H), 7.56 (s, 1H), 4.35 (dd, J = 5.1, 3.0 Hz, 2H), 4.30 – 4.25 (m, 2H), 3.63 (p, J = 6.8, 6.8, 6.8, 6.8 Hz, 1H), 2.10 (s, 3H), 1.09 (d, J = 6.8 Hz, 6H). HRMS (ESI+): calcd for C14H17NO4 (M + H)+, 264.1230; found 264.1219.

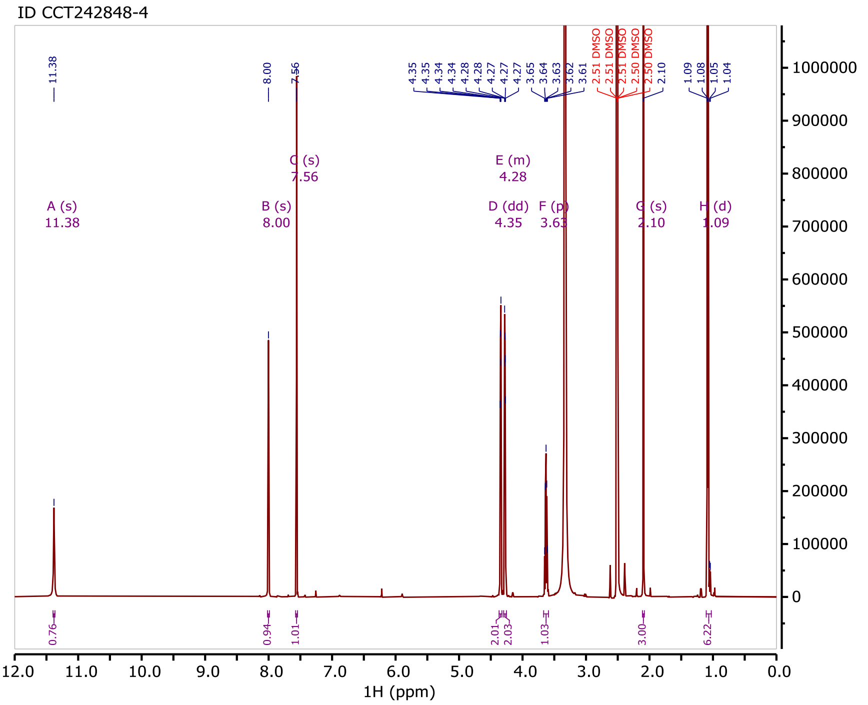

Parameter	Value
Title	ML-5245-069-CCT242848-4.10.fid
Comment	ID CCT242848-4
Probe	Z75812_0002 (CP TCI 600S3 H-C/ N-D-05 Z)
Number of Scans	32
Receiver Gain	101.0
Relaxation Delay	20.0000
Acquisition Date	2021-09-05T06:01:58
Spectrometer Frequency	600.13

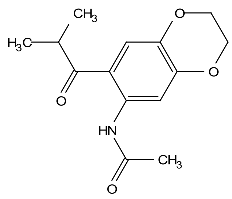

Figure S17 ^{1}H-NMR spectrum of CCT242848-4

	Parameter	Value
1	Data Path	D:\ MassHunter\ Walkup\ DataFiles\ mliu\ QT22_02137_035.d
2	Acquired Date	2022-03-23T16:21:02.841Z
3	Sample Name	QT22_02137
4	Comment	ML-242848
5	Sample Position	P1-B2

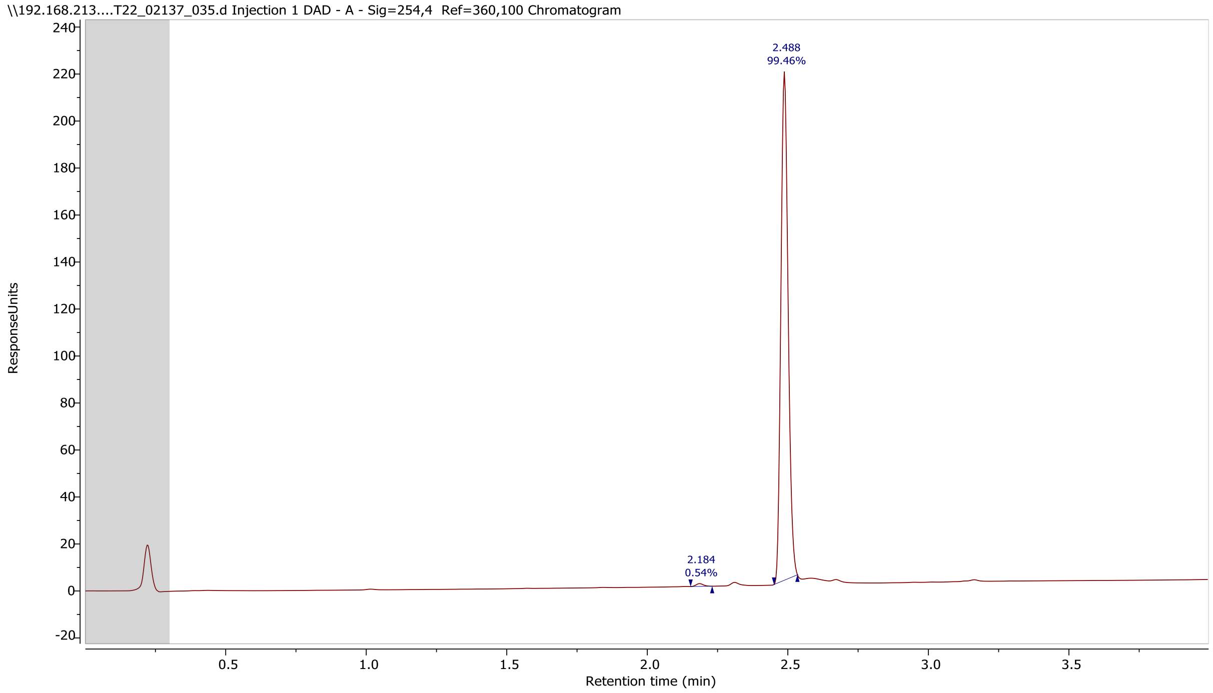

Figure S18 LC-UV trace of CCT242848-4

CCT239822-4

1H NMR (600 MHz, DMSO) δ 7.66 (s, 1H), 7.23 – 7.19 (m, 2H), 6.90 – 6.84 (m, 2H), 4.00 (q, J = 7.0, 7.0, 7.0 Hz, 2H), 3.55 (dq, J = 16.
8.2, 8.2 Hz, 2H), 3.14 (p, J = 7.4, 7.4, 7.2, 7.2 Hz, 1H), 2.48 – 2.43 (m, 1H), 2.25 (dd, J = 16.3, 9.1 Hz, 1H), 1.31 (t, J = 7.0, 7.0 Hz

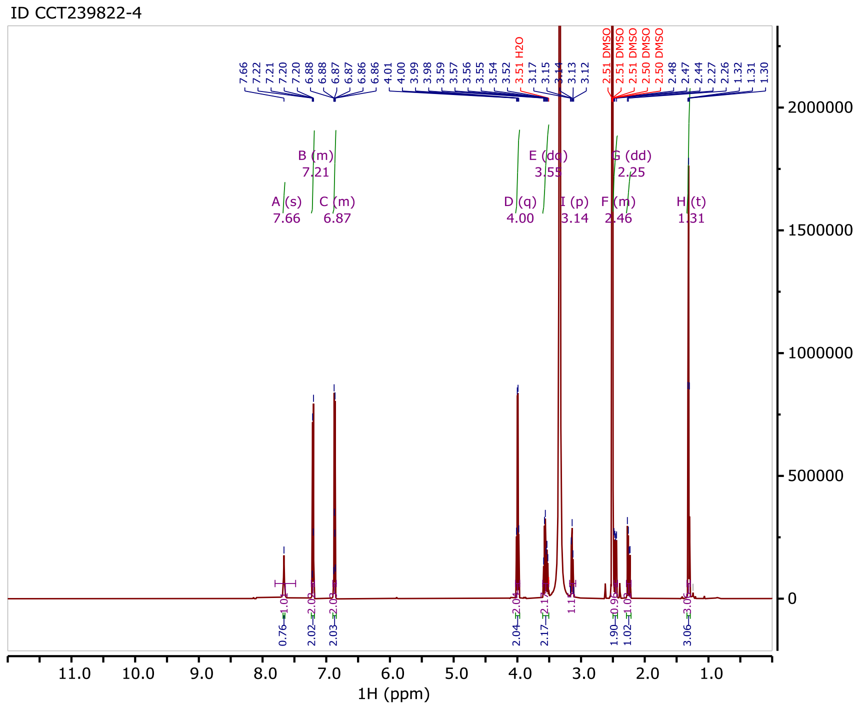

Parameter	Value
Title	ML-5245-069-CCT239822-4.10.fid
Comment	ID CCT239822-4
Probe	Z75812_0002 (CP TCI 600S3 H-C/ N-D-05 Z)
Number of Scans	32
Receiver Gain	101.0
Relaxation Delay	20.0000
Acquisition Date	2021-09-03T14:10:51
Spectrometer Frequency	600.13

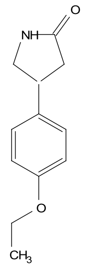

Figure S19 ¹H-NMR spectrum of CCT239822-4

	Parameter	Value
1	Data Path	D:\ MassHunter\ Walkup\ DataFiles\ mliu\ QT22_02143_042.d
2	Acquired Date	2022-03-24T09:43:39.545Z
3	Sample Name	QT22_02143
4	Comment	ML-239822
5	Sample Position	P1-A4

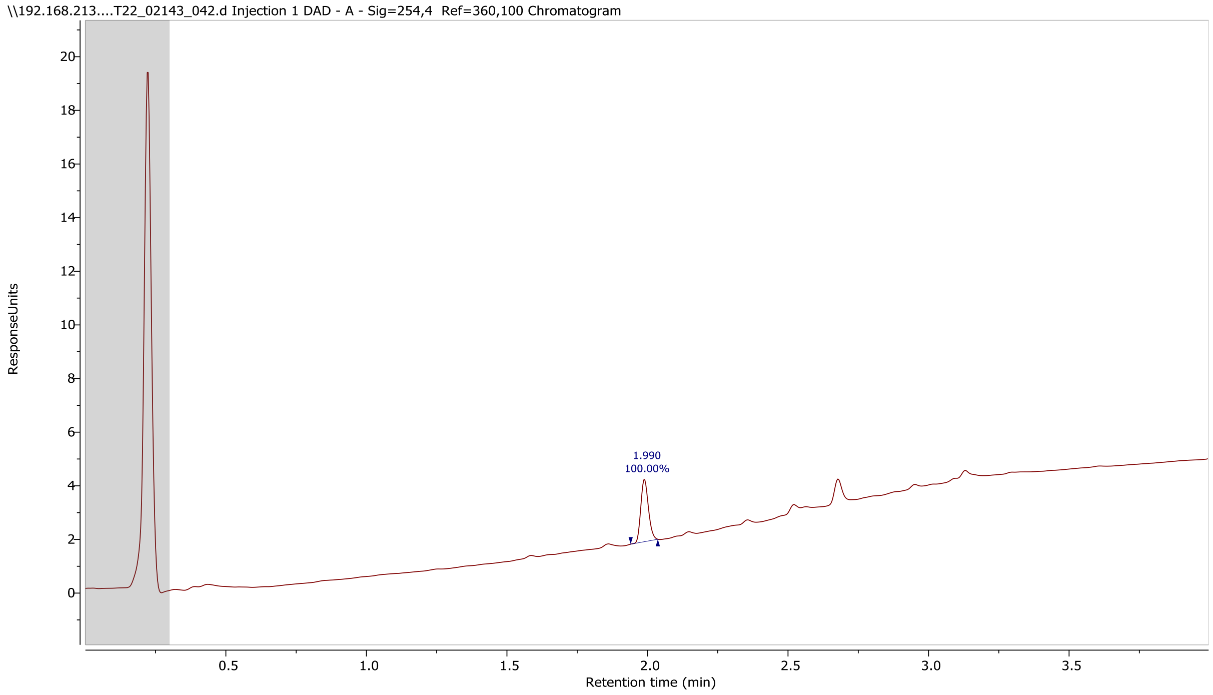

Figure S20 LC-UV trace of CCT239822-4

CCT242739-4

1H NMR (600 MHz, DMSO) δ 9.12 (d, J = 2.3 Hz, 1H), 8.70 (dd, J = 4.8, 1.5 Hz, 1H), 8.30 (dt, J = 8.0, 1.9, 1.9 Hz, 1H), 7.56 (dd, J = 8.0, 4.8 Hz, 1H), 3.51 (s, 4H), 2.42 (s, 3H), 1.64 (p, J = 6.1, 6.1, 5.7, 5.7 Hz, 2H), 1.54 (s, 4H). HRMS (ESI+): calcd for $C_{15}H_{17}N_3OS$ (M + H)+, 288.1165; found 288.1145.

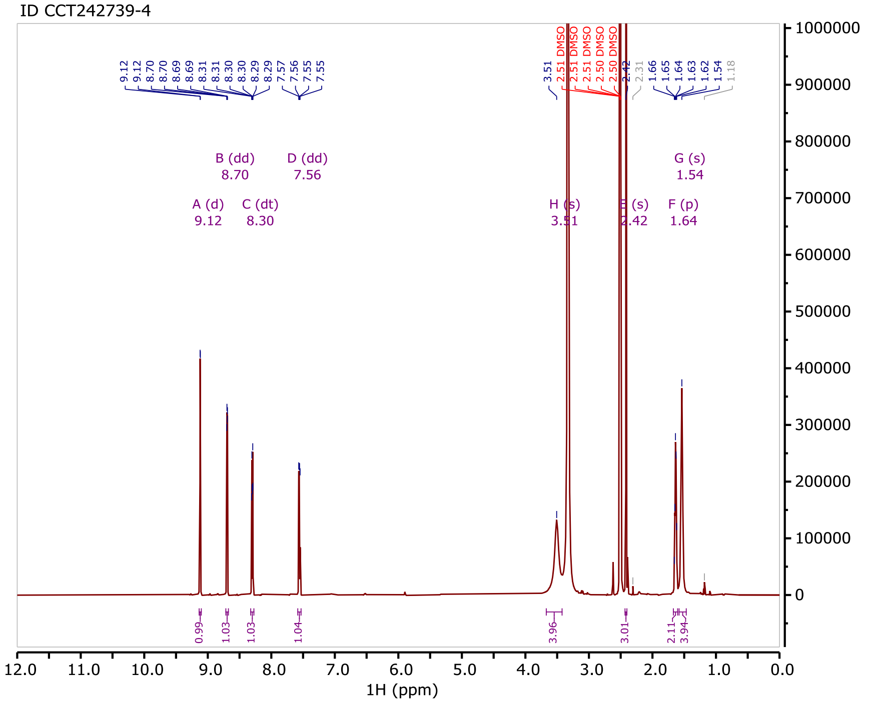

Parameter	Value
Title	ML-5245-069-CCT242739-4.10.fid
Comment	ID CCT242739-4
Probe	Z75812_0002 (CP TCI 600S3 H-C/ N-D-05 Z)
Number of Scans	32
Receiver Gain	101.0
Relaxation Delay	20.0000
Acquisition Date	2021-09-05T05:39:24
Spectrometer Frequency	600.13

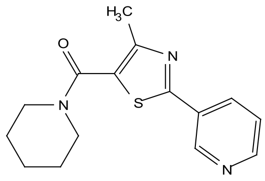

Figure S21 ^{1}H-NMR spectrum of CCT232739-4

	Parameter	Value
1	Data Path	D:\ MassHunter\ Walkup\ DataFiles\ mliu\ QT22_02144_044.d
2	Acquired Date	2022-03-24T09:54:23.187Z
3	Sample Name	QT22_02144
4	Comment	ML-242739
5	Sample Position	P3-A3

\\192.168.213....T22_02144_044.d Injection 1 DAD - A - Sig=254,4 Ref=360,100 Chromatogram

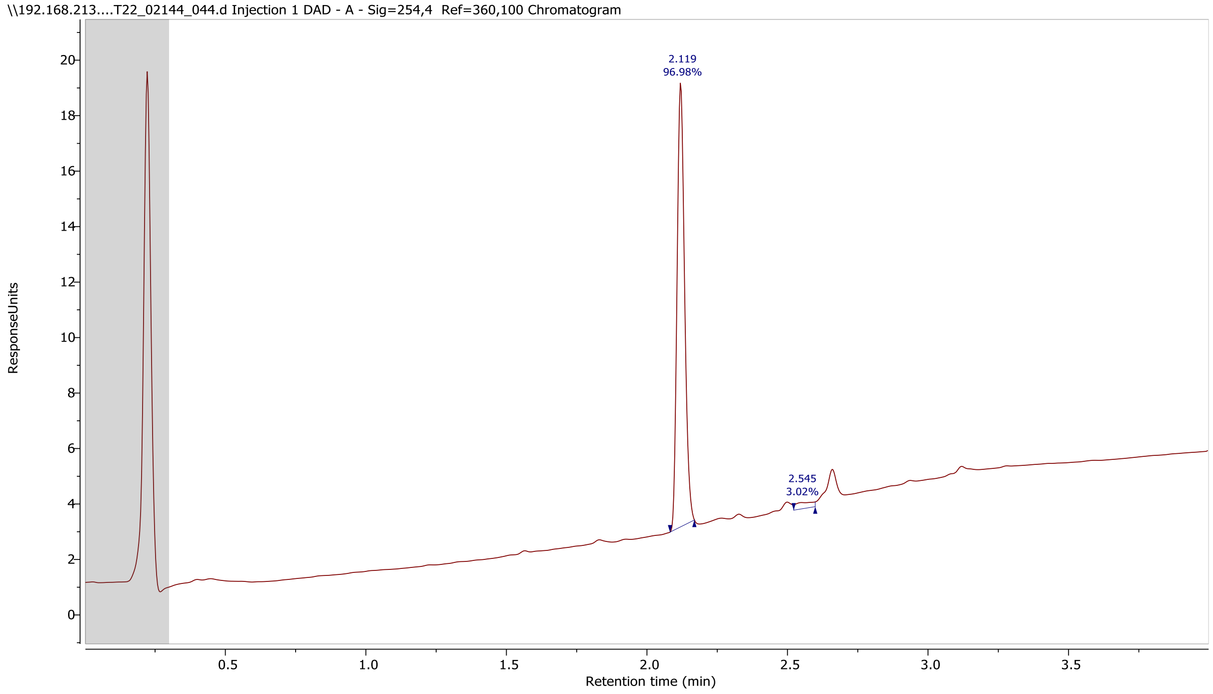

Figure S22 LC-UV trace of CCT232739-4

CCT224736-5

1H NMR (600 MHz, DMSO) δ 10.55 (s, 1H), 7.81 (d, J = 8.4 Hz, 1H), 7.45 (s, 1H), 7.33 (dd, J = 8.3, 1.8 Hz, 1H), 5.37 (s, 2H), 3.16 (s, 3H).
HRMS (ESI+): calcd for C9H9NO4S (M + H)+, 228.0325; found 228.0313.

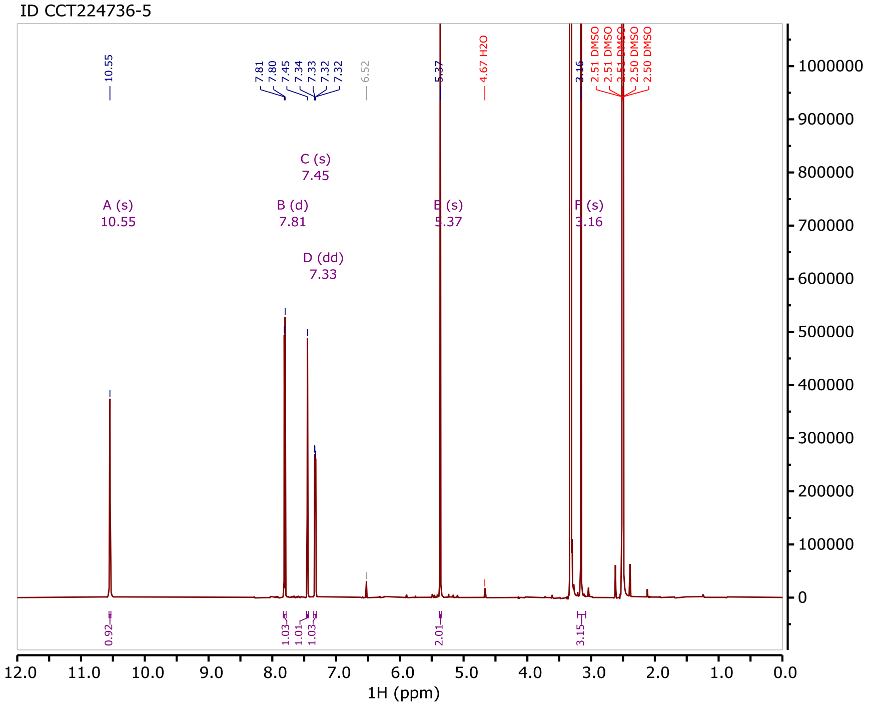

Parameter	Value
Title	ML-5245-069-CCT224736-5.10.fid
Comment	ID CCT224736-5
Probe	Z75812_0002 (CP TCI 600S3 H-C/ N-D-05 Z)
Number of Scans	32
Receiver Gain	101.0
Relaxation Delay	20.0000
Acquisition Date	2021-09-05T04:32:23
Spectrometer Frequency	600.13

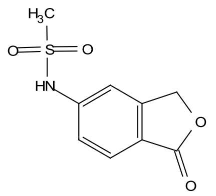

Figure S23 ^{1}H-NMR spectrum of CCT224736-5

	Parameter	Value
1	Data Path	D:\ MassHunter\ Walkup\ DataFiles\ mliu\ QT22_02151_053.d
2	Acquired Date	2022-03-24T11:04:46.536Z
3	Sample Name	QT22_02151
4	Comment	ML-224736
5	Sample Position	P1-A9

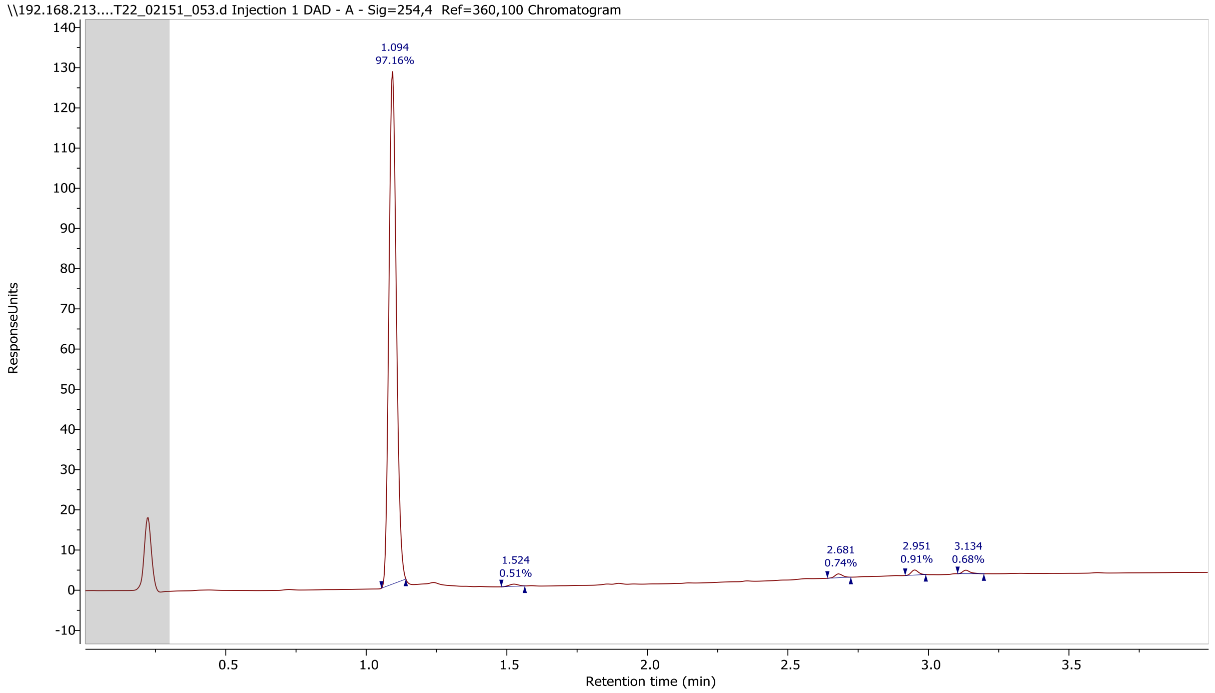

Figure S24 LC-UV trace of CCT224736-5

CCT228155-5

1H NMR (600 MHz, DMSO) δ 7.34 (d, J = 2.2 Hz, 1H), 7.09 – 7.01 (m, 2H), 4.67 (s, 2H), 4.00 – 3.95 (m, 2H), 2.60 (q, J = 8.0, 8.0, 7.2 Hz, 2H), 1.86 (p, J = 7.2, 7.2, 7.2, 7.2 Hz, 2H). HRMS (ESI+): calcd for C12H11ClN2O2 (M + H)+, 251.0582; found 251.0567.

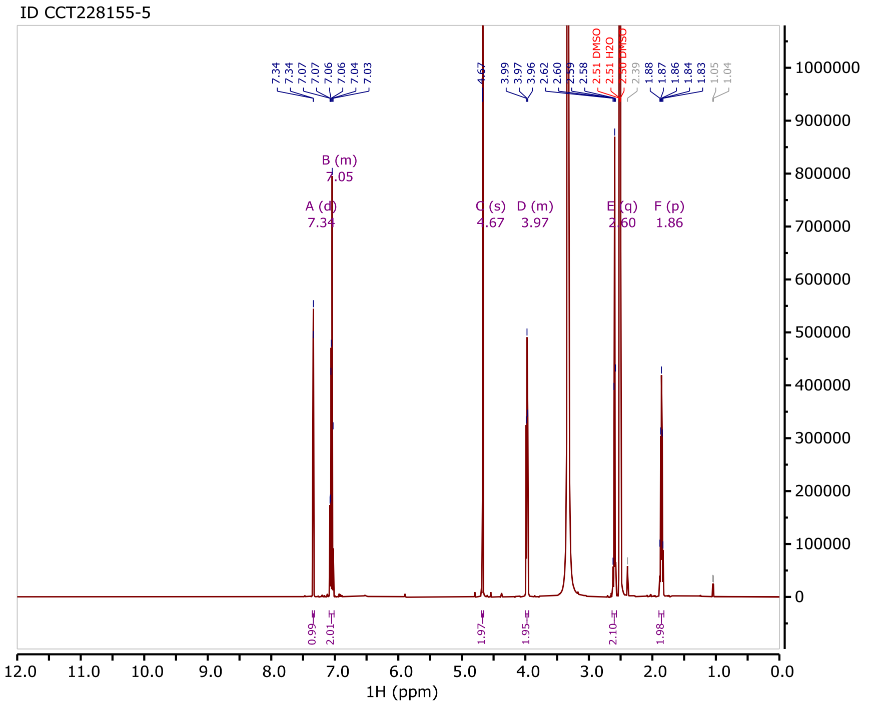

Parameter	Value
Title	ML-5245-069-CCT228155-5.10.fid
Comment	ID CCT228155-5
Probe	Z75812_0002 (CP TCI 600S3 H-C/ N-D-05 Z)
Number of Scans	32
Receiver Gain	101.0
Relaxation Delay	20.0000
Acquisition Date	2021-09-05T06:24:48
Spectrometer Frequency	600.13

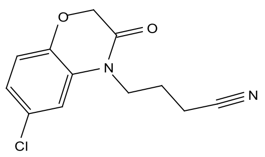

Figure S25 ^{1}H-NMR spectrum of CCT228155-5

	Parameter	Value
1	Data Path	D:\ MassHunter\ Walkup\ DataFiles\ mliu\ QT22_02152_055.d
2	Acquired Date	2022-03-24T11:15:32.428Z
3	Sample Name	QT22_02152
4	Comment	ML-228155
5	Sample Position	P3-A5

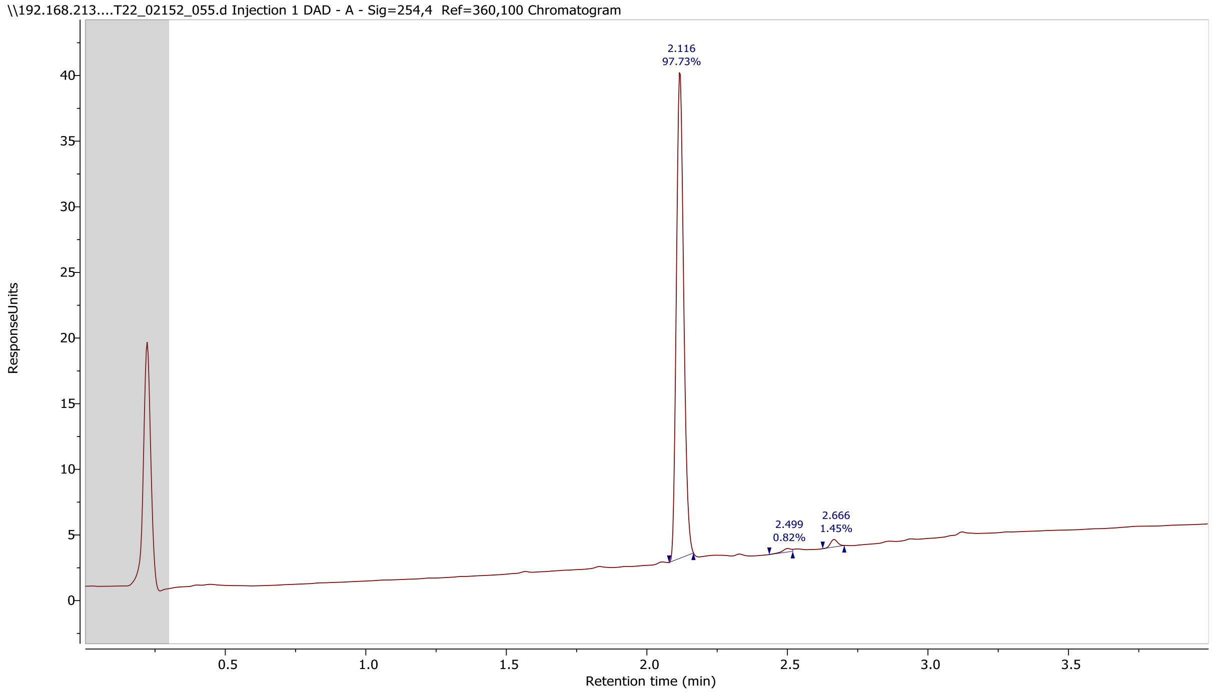

Figure S26 LC-UV trace of CCT228155-5

CCT240545-4

1H NMR (600 MHz, DMSO) δ 8.26 (t, J = 5.7, 5.7 Hz, 1H), 7.60 (d, J = 8.6 Hz, 2H), 6.91 – 6.79 (m, 4H), 6.54 (d, J = 8.6 Hz, 2H), 5.63 (s, 2H), 4.30 (ddd, J = 11.1, 9.2, 2.2 Hz, 2H), 3.96 (dd, J = 11.9, 7.4 Hz, 1H), 3.54 (dt, J = 13.5, 5.8, 5.8 Hz, 1H), 3.45 (dt, J = 13.5, 6.0, 6.0 Hz, 1H). HRMS (ESI+): calcd for $C_{16}H_{16}N_2O_3$ (M + H)+, 285.1234; found 285.1209.

ID CCT240545-4

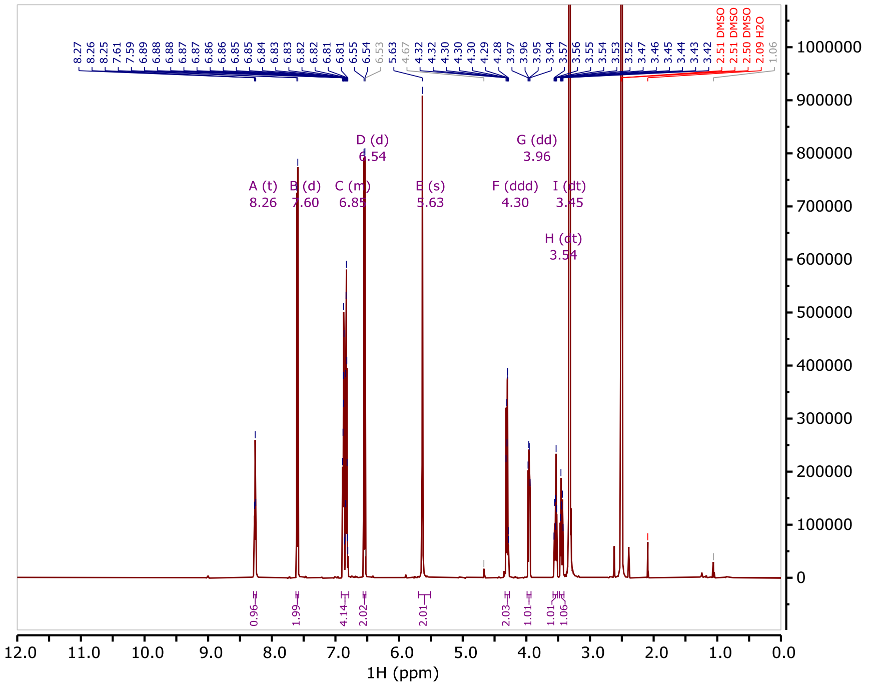

Parameter	Value
Title	ML-5245-069-CCT240545-4.10.fid
Comment	ID CCT240545-4
Probe	Z75812_0002 (CP TCI 600S3 H-C/ N-D-05 Z)
Number of Scans	32
Receiver Gain	101.0
Relaxation Delay	20.0000
Acquisition Date	2021-09-05T04:54:46
Spectrometer Frequency	600.13

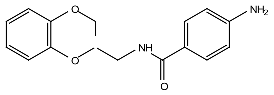

Figure S27 ^{1}H-NMR spectrum of CCT240545-4

	Parameter	Value
1	Data Path	D:\ MassHunter\ Walkup\ DataFiles\ mliu\ QT22_02152_056.d
2	Acquired Date	2022-03-24T11:21:06.009Z
3	Sample Name	QT22_02152
4	Comment	ML-228155
5	Sample Position	P3-A5

\\192.168.213....T22_02152_056.d Injection 1 DAD - A - Sig=254,4 Ref=360,100 Chromatogram

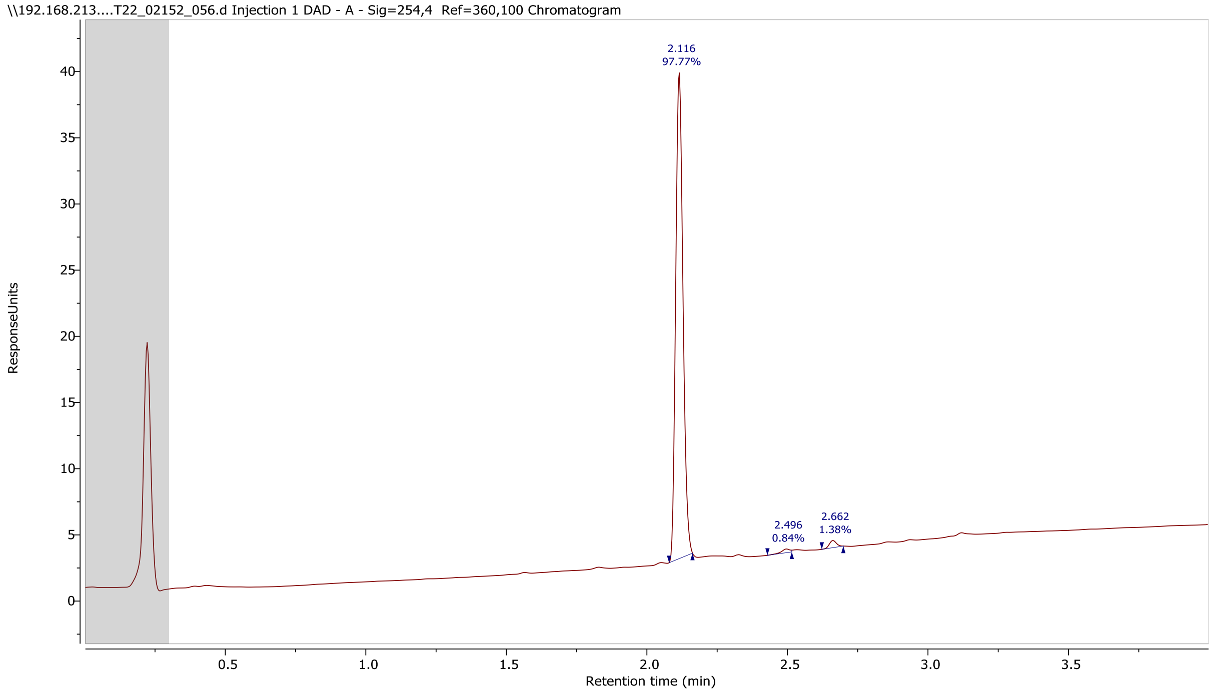

Figure S28 LC-UV trace of CCT240545-4

CCT242858-4

1H NMR (600 MHz, DMSO) δ 9.92 (s, 1H), 9.30 (s, 1H), 7.08 (t, J = 7.8, 7.8 Hz, 1H), 6.67 (s, 1H), 6.62 – 6.57 (m, 3H), 6.48 (s, 1H), 4.15 – 4.11 (m, 1H), 3.72 (s, 3H), 3.63 (s, 3H), 2.83 (dd, J = 16.0, 6.8 Hz, 1H), 2.56 (dd, J = 16.0, 4.6 Hz, 1H).
HRMS (ESI+): calcd for $C_{17}H_{17}NO_4$ $(M + H)^+$, 300.1230; found 300.1214.

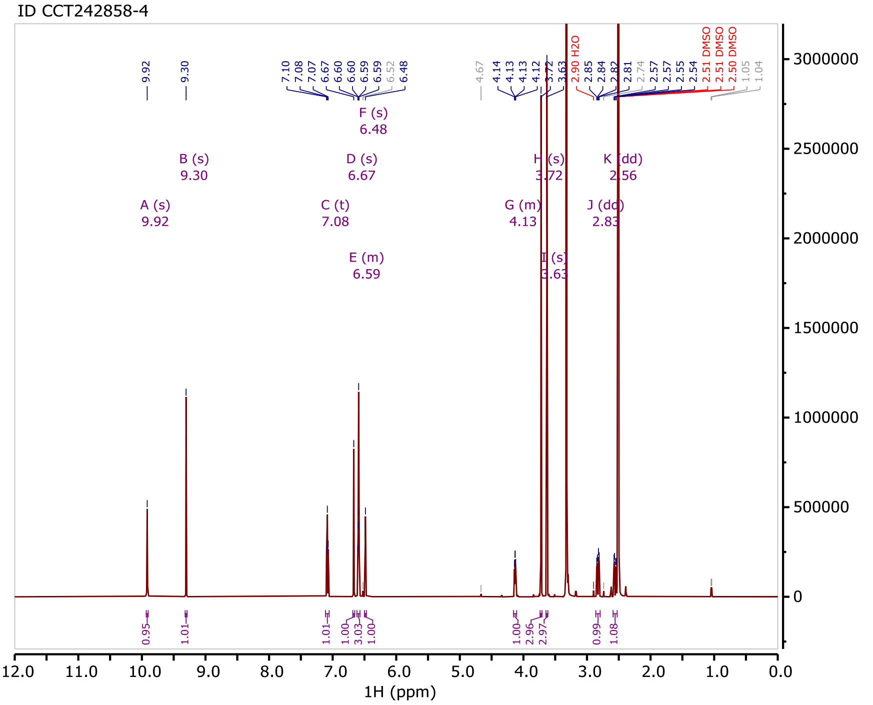

Parameter	Value
Title	ML-5245-069-CCT242858-4.10.fid
Comment	ID CCT242858-4
Probe	Z75812_0002 (CP TCI 600S3 H-C/ N-D-05 Z)
Number of Scans	32
Receiver Gain	101.0
Relaxation Delay	20.0000
Acquisition Date	2021-09-05T06:46:43
Spectrometer Frequency	600.13

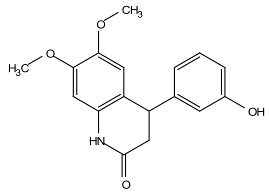

Figure S29 ^{1}H-NMR spectrum of CCT242858-4

	Parameter	Value
1	Data Path	D:\ MassHunter\ Walkup\ DataFiles\ mliu\ QT22_02157_063.d
2	Acquired Date	2022-03-24T13:49:48.451Z
3	Sample Name	QT22_02157
4	Comment	ML-242858
5	Sample Position	P1-B4

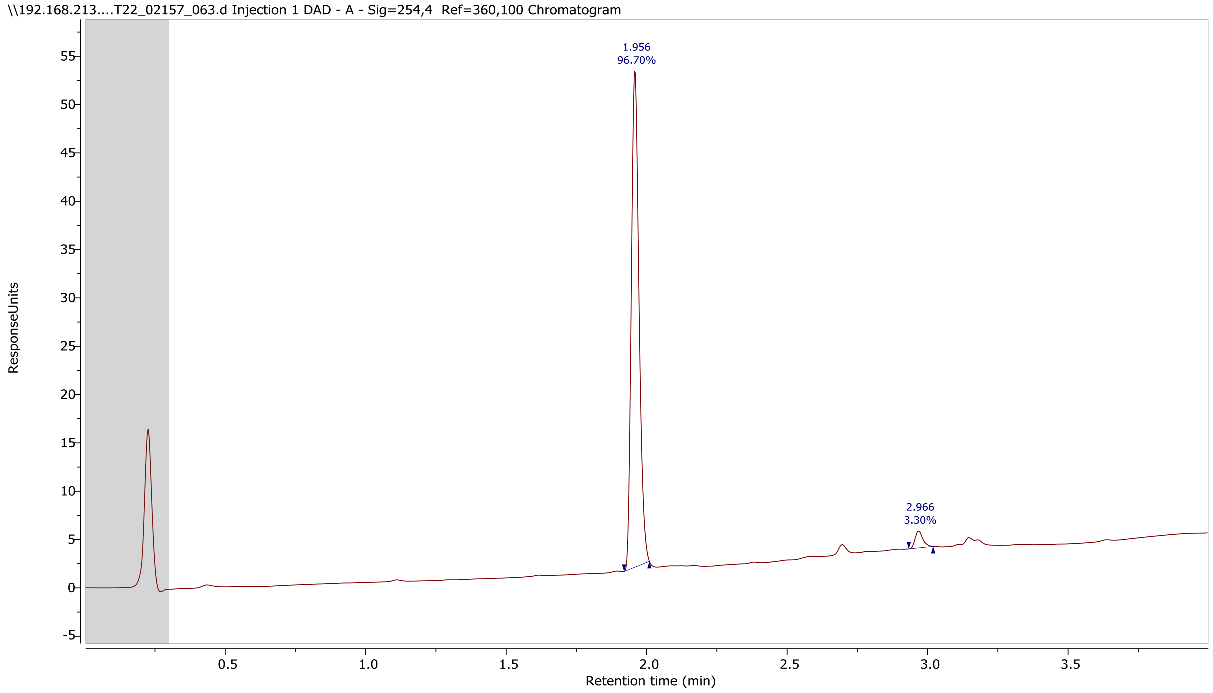

Figure S30 LC-UV trace of CCT242858-4

K_d curve fitting data for the top ten fragments identified in fragment screening campaign against CRBN/DDB1 complex

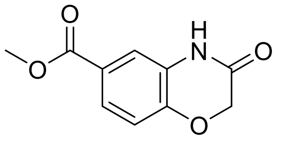

CCT240569
KD 72 uM

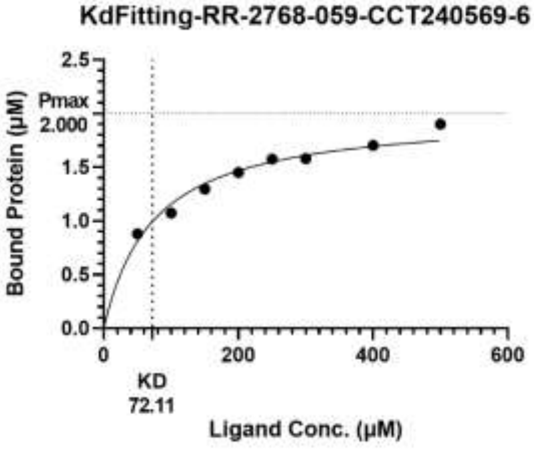

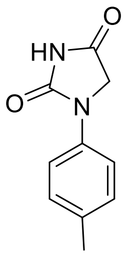

CCT010354
KD 122 uM

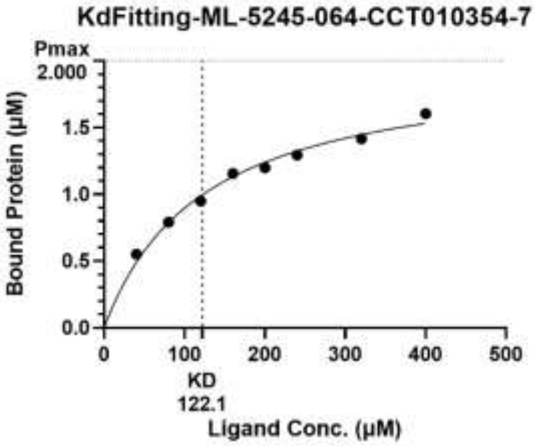

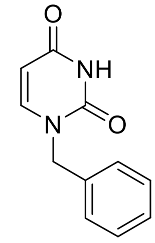

CCT240207
KD 85.9 uM

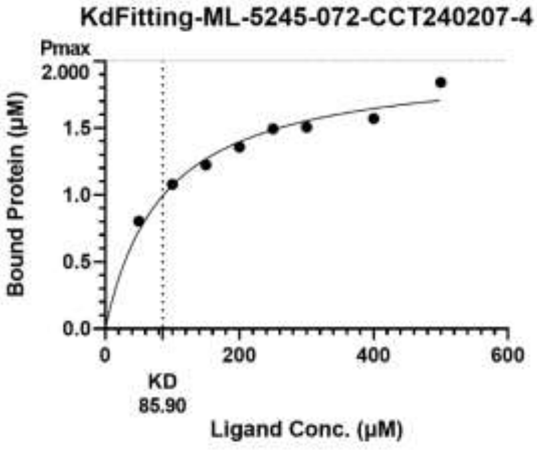

Figure S31 Kd fitting curve data from R2KD assay for CCT240569, CCT010354 and CCT240207

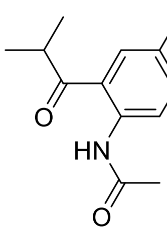

CCT242848
KD 532 uM

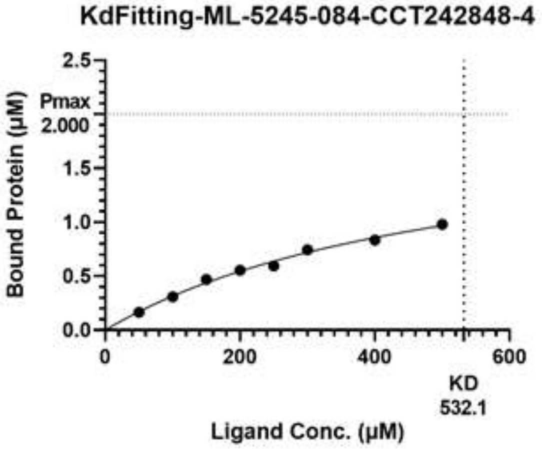

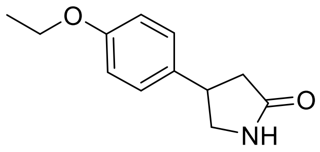

CCT239822
KD 532 uM

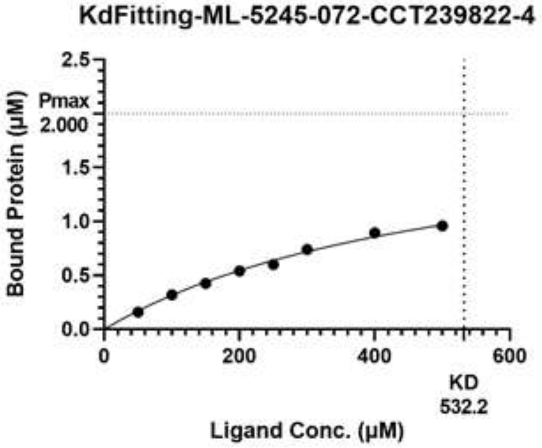

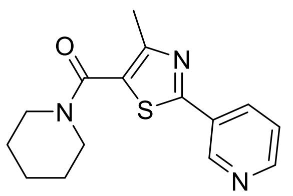

CCT242739
KD 695 uM

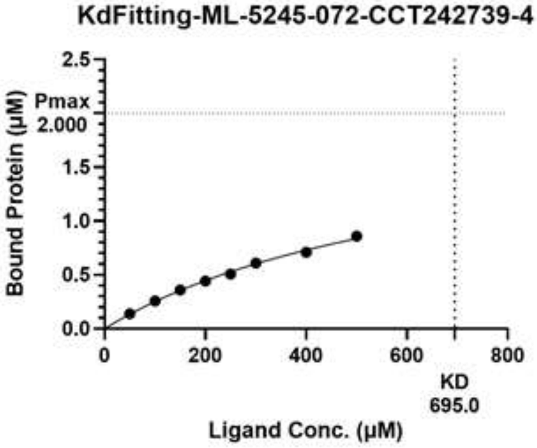

Figure S32 Kd fitting curve data from R2KD assay for CCT242848, CCT239822 and CCT242739

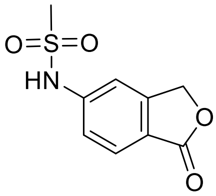

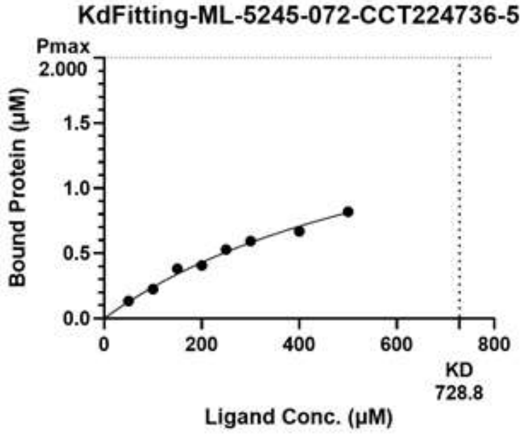

CCT224736
KD 729 uM

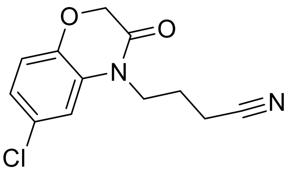

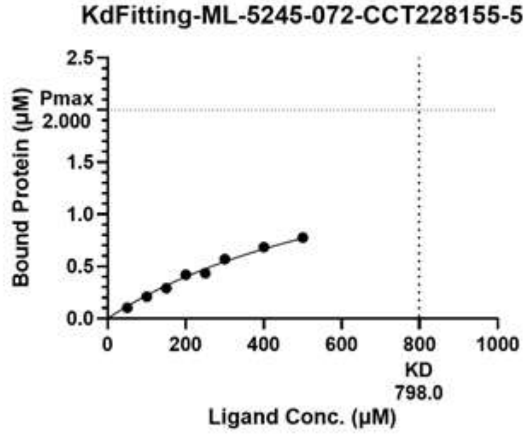

CCT228155
KD 798 uM

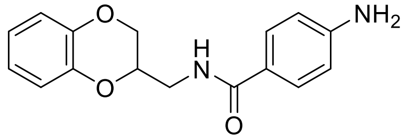

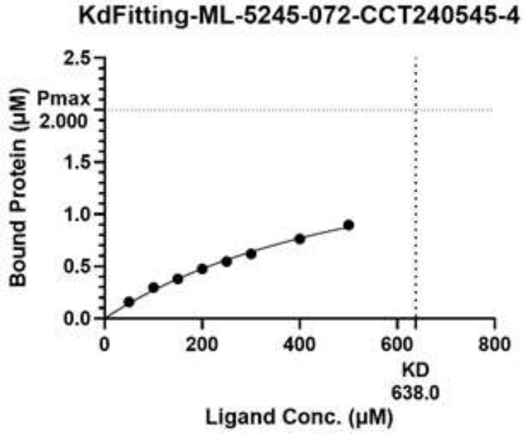

CCT240545
KD 638 uM

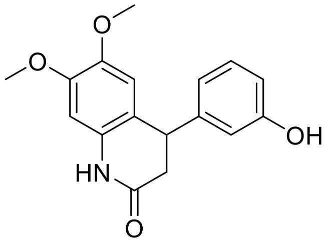

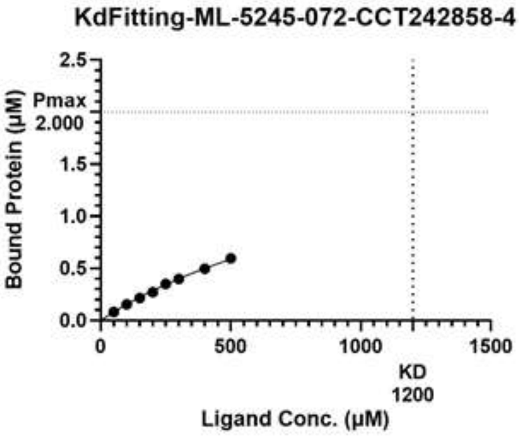

CCT242858
KD 1200 uM

Figure S33 Kd fitting curve data from R2KD assay for CCT224736, CCT228155, CCT240545 and CCT242858.

A correlation plot between pK_d and pK_i using data presented in Table 1.

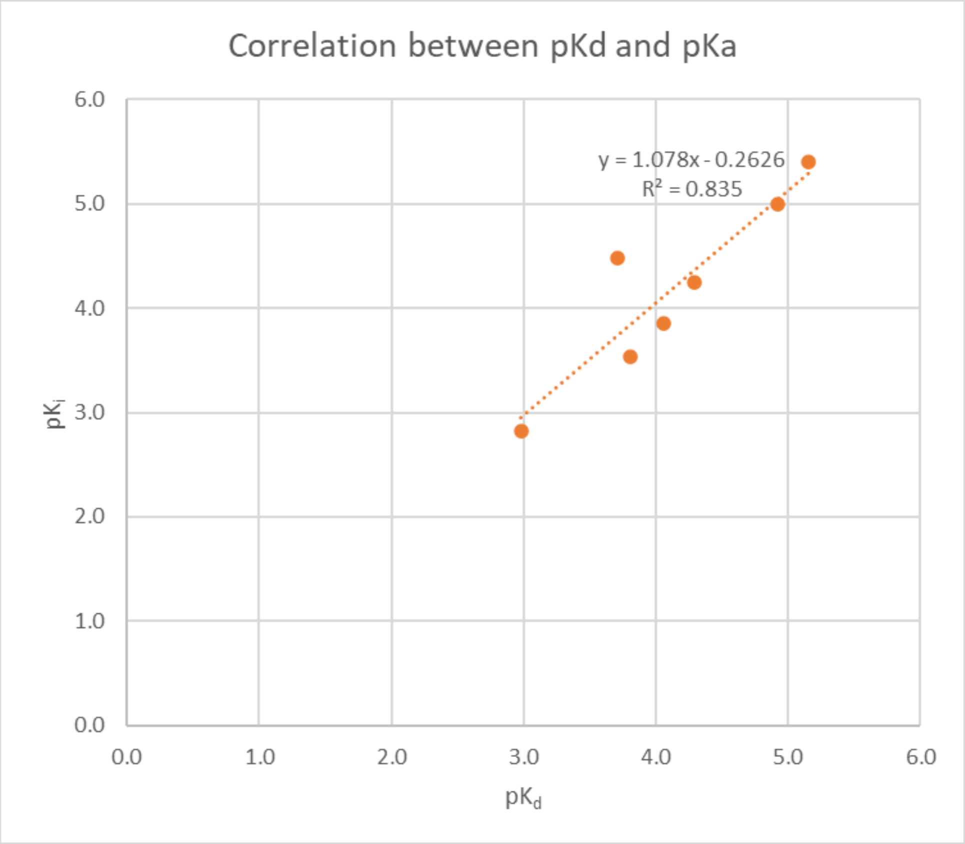

Figure S34 a correlation plot between pK_d values and pK_i values presented in Table 1 in the manuscript.
